# A transcriptional regulatory mechanism of genes in the tricarboxylic acid cycle in the heart

**DOI:** 10.1016/j.jbc.2024.107677

**Published:** 2024-08-14

**Authors:** Samta Veera, Fan Tang, Youssef Mourad, Samuel Kim, Tong Liu, Hong Li, Yunjue Wang, Junco S. Warren, Jiyeon Park, Carter Van, Junichi Sadoshima, Shin-ichi Oka

**Affiliations:** 1 Department of Cell Biology and Molecular Medicine, Rutgers New Jersey Medical School, Newark, New Jersey, USA; 2 Center for Advanced Proteomics Research, Department of Microbiology, Biochemistry, and Molecular Genetics, Rutgers New Jersey Medical School and Cancer Institute of New Jersey, Newark, New Jersey, USA; 3 Metabolomics Shared Resource, Rutgers Cancer Institute of New Jersey, New Brunswick, New Jersey, USA; 4 Fralin Biomedical Research Institute at Virginia Tech Carilion, Virginia Tech, Roanoke, Virginia, USA; 5 Precision Medicine Research Center, College of Medicine, The Catholic University of Korea, Seoul, Republic of Korea

**Keywords:** mitochondria, tricarboxylic acid (TCA) cycle, transcription, PGC-1α, ERR, Nrf1, Gabpa, YY1

## Abstract

The tricarboxylic acid (TCA) cycle plays a crucial role in mitochondrial ATP production in the healthy heart. However, in heart failure, the TCA cycle becomes dysregulated. Understanding the mechanism by which TCA cycle genes are transcribed in the healthy heart is an important prerequisite to understanding how these genes become dysregulated in the failing heart. PPARγ coactivator 1α (PGC-1α) is a transcriptional coactivator that broadly induces genes involved in mitochondrial ATP production. PGC-1α potentiates its effects through the coactivation of coupled transcription factors, such as estrogen-related receptor (ERR), nuclear respiratory factor 1 (Nrf1), GA-binding protein-a (Gabpa), and Yin Yang 1 (YY1). We hypothesized that PGC-1α plays an essential role in the transcription of TCA cycle genes. Thus, utilizing localization peaks of PGC-1α to TCA cycle gene promoters would allow the identification of coupled transcription factors. PGC-1α potentiated the transcription of 13 out of 14 TCA cycle genes, partly through ERR, Nrf1, Gabpa, and YY1. ChIP-sequencing showed PGC-1α localization peaks in TCA cycle gene promoters. Transcription factors with binding elements that were found proximal to PGC-1α peak localization were generally essential for the transcription of the gene. These transcription factor binding elements were well conserved between mice and humans. Among the four transcription factors, ERR and Gabpa played a major role in potentiating transcription when compared to Nrf1 and YY1. These transcription factor-dependent PGC-1α recruitment was verified with Idh3a, Idh3g, and Sdha promoters with DNA binding assay. Taken together, this study clarifies the mechanism by which TCA cycle genes are transcribed, which could be useful in understanding how those genes are dysregulated in pathological conditions.

The mitochondrion is an energy-generating organelle that produces the ATP required for normal cardiomyocyte function. The tricarboxylic acid (TCA) cycle plays a crucial role in mitochondrial ATP production (1). The TCA cycle participates in both anabolism and catabolism reactions; thus, it is considered a signaling hub, as its metabolites play a crucial role in a variety of essential processes, including maintaining cellular homeostasis, post-translational protein modification, DNA methylation, and chromatin remodeling (2).

Genetic defects affecting TCA cycle enzymes are well associated with various disorders, such as severe neurological damage and cancer (3). Defects in TCA cycle enzymes lead to the accumulation of upstream TCA cycle metabolites, which are found to be associated with various human cancers (4). Several TCA cycle genes are downregulated in pressure overload-induced heart failure mouse models, such as Sdha, Idh3a, and Fh1 (5, 6). To clarify the mechanism responsible for expressional changes of TCA cycle genes in heart failure models, it is necessary to clarify the mechanism by which these genes are transcribed in normal healthy conditions.

There are many possible transcription factors involved in the transcription of nucleus-encoded mitochondrial genes. Examples include nuclear receptors, nuclear respiratory factor 1 (Nrf1), GA-binding protein-a (Gabpa), Yin Yang 1 (YY1), and c-myc (7). However, whether and how these transcription factors transcribe TCA cycle genes has not been clarified. Due to the number of factors possibly involved, it presents a difficulty in testing their role in transcription and identifying the binding element in all TCA cycle genes. PPARγ coactivator 1α (PGC-1α) is a transcriptional coactivator that broadly induces metabolic genes (8). Several transcription factors commonly use PGC-1α for transcriptional activation such as estrogen-related receptor (ERR), Nrf1, Gabp, YY1, peroxisome proliferator-activated receptors (PPAR), FoxO1, and myocyte enhancer factor-2C (Mef2c) (9). We took advantage of this molecular property of PGC-1α in this study. Namely, if PGC-1α essentially coactivates transcription of TCA cycle genes, determination of PGC-1α localization peaks in their promoters would help in the identification of a critical transcription factor and its binding element. With this strategy, this study elucidated a fundamental mechanism of how TCA cycle genes are transcribed in the heart.

## Results

### Stress response in the TCA cycle genes

**Figure 1:**
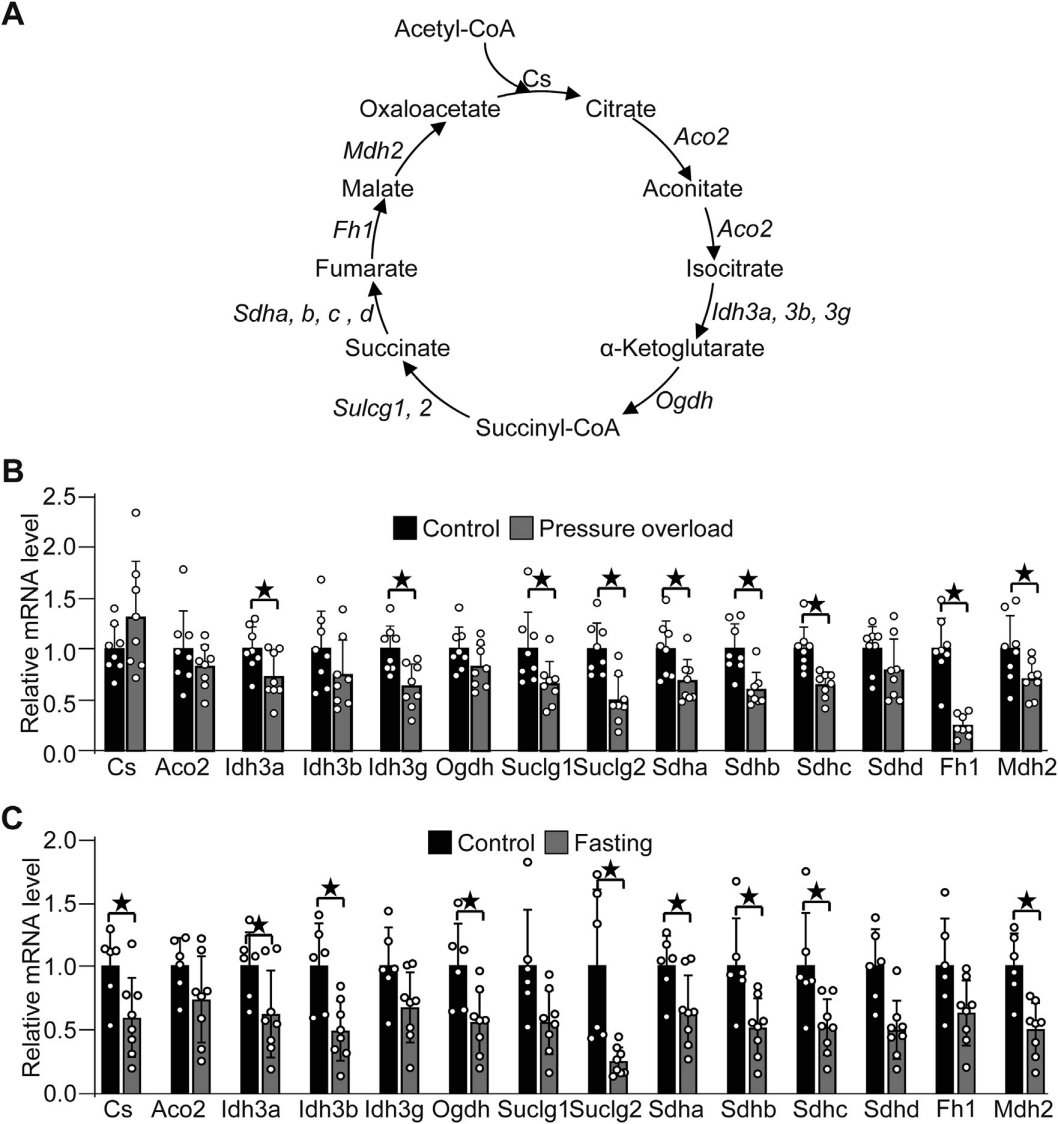
**Regulation of TCA cycle genes in stress conditions.***A*, a schematic representation of the TCA cycle. The TCA cycle consists of eight enzymes composed of 14 genes involved in sequential linear steps required for mitochondrial energy production. Abbreviations of the enzymes are as follows. Aco2, Aconitase 2; Cs, Citrate Synthase; Fh1, Fumarate Hydratase 1; Idh3, Isocitrate Dehydrogenase; Mdh2, Malate Dehydrogenase 2; Ogdh, Oxoglutarate Dehydrogenase; Sdh, Succinate Dehydrogenase; and Suclg, Succinate-CoA Ligase. *B-C*, the expression of TCA cycle genes in response to pressure overload (2 weeks) (*B*) and fasting (*C*). n = 8(A) and 6 to 8(B). ★*p* < 0.05 vs control mice.

The TCA cycle plays a central role in mitochondrial energy production. However, how those genes are regulated under stress conditions is not fully understood. Here, we used the pressure overload (PO)-induced heart failure model as a pathological stress and fasting as a physiological stress. There are eight enzymes composed of 14 genes in the TCA cycle (Fig. 1*A*). As shown in Figure 1*B*, PO significantly downregulated several TCA cycle genes, including Idh3a, Idh3g, Suclg1, Suclg2, Sdha, Sdhb, Sdhc, Fh1, and Mdh2. There is a trend showing upregulation of CS and downregulation of Aco2 and Ogdh. As shown in Figure 1*C*, fasting significantly downregulated Cs, Idh3a, Idh3b, Ogdh, Suclg3, Sdha, Sdhb, Sdhc, and Mdh2, whereas the other genes showed a trend of downregulation. These results suggest that many, if not all, TCA cycle genes are suppressed in response to stresses, which could be a maladaptive response in the failing heart leading to insufficient energy production, but an adaptive response in fasting to minimize nutrient usage.

### PGC-1α direct target genes in the TCA cycle

**Figure 2:**
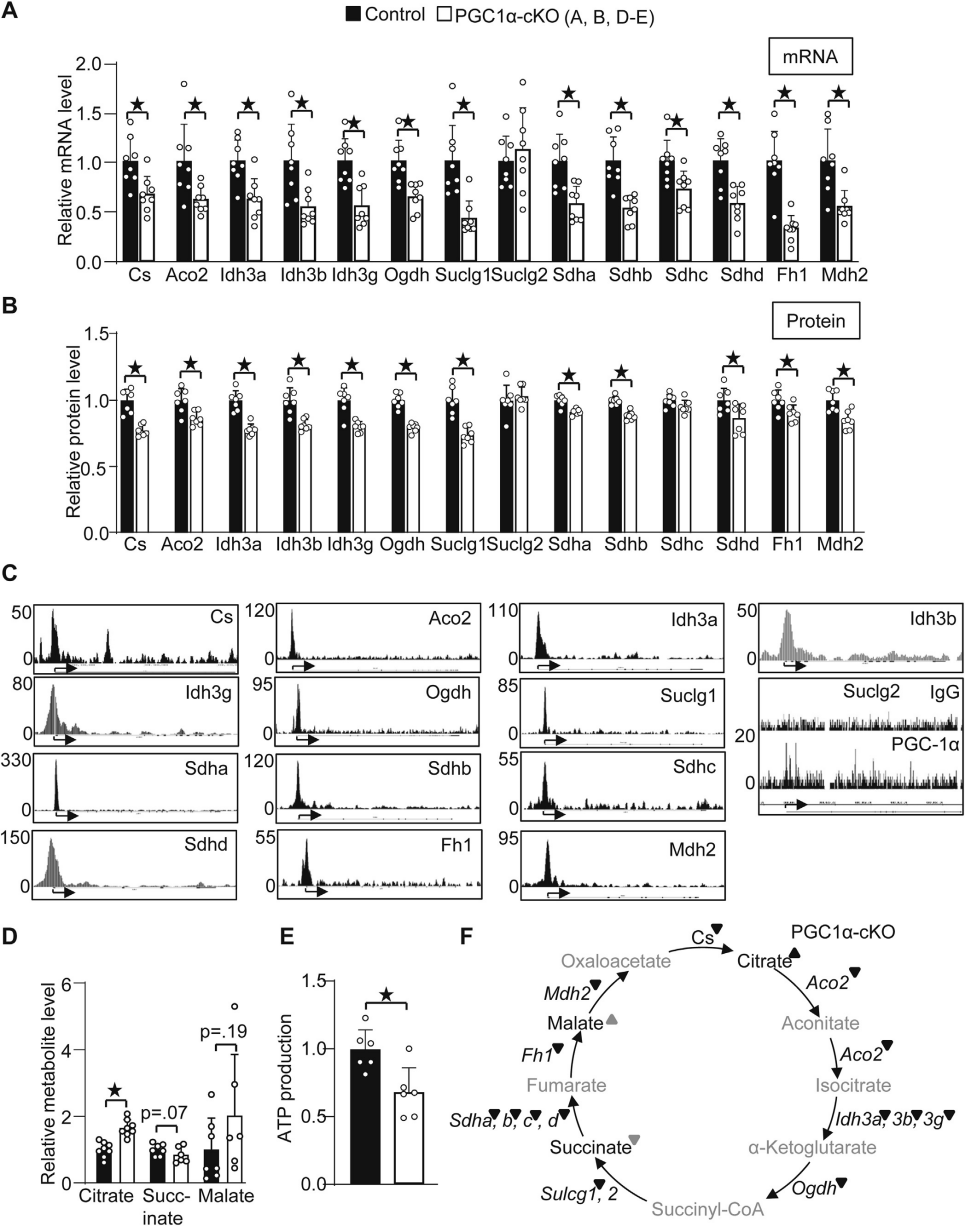
**Many, if not all, TCA cycle genes are PGC-1α targets.***A* and *B*, the levels of TCA cycle genes at mRNA (*A*) and protein (*B*) in PGC-1αcKO mice. *C*, ChIP-seq shows PGC-1α localization in the promoter regions of 13 out of 14 TCA cycle genes. These ChIP-seq images magnifying a promoter region are reused in Figure 3*A*. *D*, the levels of citrate, succinate and malate metabolites in PGC-1αcKO mice, detected with enzymatic assay (citrate) and metabolome analysis (succinate and malate). *E*, decrease in ATP production in PGC-1αcKO mice. n = 8(*A*), 7(*B*), 6 to 7(*D*) and 6(*E*). (*F*) Schematic representation of how the TCA cycle is regulated. ▼: Downregulation at mRNA or protein levels. ▼ or ▲: Trend of down or upregulation. Metabolites not detected is shown by gray. ★*p* < 0.05 vs control mice. PGC-1α, PPARγ coactivator 1α.

To understand how TCA cycle genes are transcriptionally regulated in stress conditions, it is a prerequisite to elucidate the mechanism by which these genes are transcribed in normal healthy conditions. PGC-1α broadly induces genes involved in mitochondrial energy production, which may include TCA cycle genes. If so, the determination of PGC-1α localization in the promoters would help in the identification of the transcription factors and their binding element that essentially transcribe the TCA cycle genes. To investigate if PGC-1α mediates transcription of TCA cycle genes in the heart, the mRNA levels of these genes were examined in cardiac-specific PGC-1α knockout (PGC-1αcKO) mice. Besides Suclg2, all TCA cycle genes were downregulated in PGC-1αcKO mice (Fig. 2*A*). To investigate if PGC-1α maintains TCA cycle genes at protein levels in an unbiased manner, proteome analyses were performed by LC-MS (Liquid chromatography-mass spectrometry) with PGC-1αcKO mice. As shown in Figure 2*B*, all 14 proteins were successfully detected in the heart. Many TCA cycle proteins were significantly downregulated in PGC-1αcKO mice, including Cs, Aco2, Idh3a, Idh3b, Idh3g, Ogdh, Suclg1, Sdha, Sdhb, Sdhd, Fh1, and Mdh2. There was a trend showing a downregulation of Sdhc. The expression of Suclg2 was not changed in PGC-1αcKO mice. Thus, PGC-1α essentially potentiates transcription of 13 among 14 TCA cycle genes. To identify genomic localization of PGC-1α in TCA cycle genes, ChIP-seq with anti-PGC-1α antibody was performed in wild-type mice. As shown in Figure 2*C*, the peaks of PGC-1α localization were observed in promoter regions of the TCA cycle genes. As is consistent with no changes in Suclg2 (Fig. 2, *A* and *B*), peaks of PGC-1α in Suclg2 promoters were faint and small compared to that observed in other promoters. Thus, Suclg2 may not be a direct target gene of PGC-1α. To investigate the extent to which PGC-1α essentially maintains TCA cycle function, metabolome analyses were performed. Metabolome analysis detected two metabolites, succinate, and malate, in the TCA cycle. Succinate was found to be decreased in PGC-1acKO mice while malate was found to be increased (Fig. 2*D*). Since metabolome analyses detected only two metabolites, we have measured the levels of citrate. A significant accumulation of citrate was observed in PGC-1αcKO mice (Fig. 2*D*). To investigate the functional significance of PGC-1α in the TCA cycle, ATP production was measured with mitochondria isolated from PGC-1αcKO mice and incubated with TCA cycle substrates including pyruvate and malate. Mitochondrial ATP production was inhibited in PGC-1α cKO mice (Fig. 2*E*). How the TCA cycle is regulated in PGC-1αcKO mice is summarized in Figure 2*F*. These results suggest that PGC-1α maintains TCA cycle function through transcription of the genes.

### Possible transcription factors recruiting PGC-1α to the TCA cycle gene promoters

**Figure 3:**
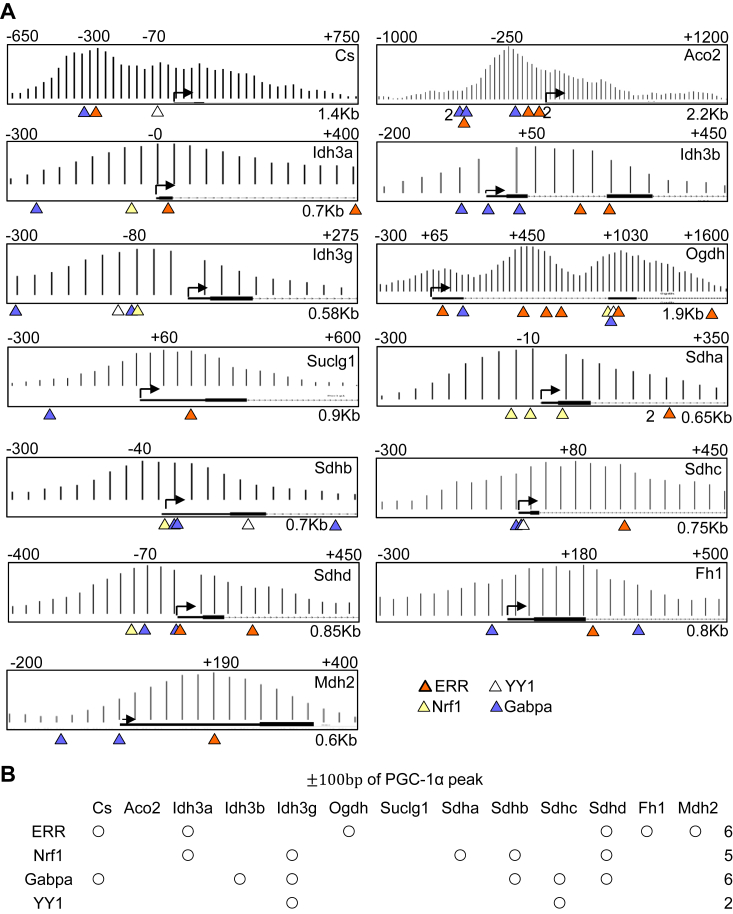
**Possible binding elements of ERR, YY1, Nrf1, and Gabpa in TCA cycle gene promoters.***A*, a bioinformatics analysis with transcription factor binding profiling identifies possible binding elements of ERR, NRf1, YY1, and Gabpa in TCA cycle gene promoters. Distance (bp) from the TSS and the peak PGC-1α location relative to the TSS are shown on the *top*, whereas the full genomic size is shown in the *right bottom* corner of ChIP-seq images. The ChIP-seq images are reused in Figure 4, Figure 6 and 6*D*, S1, S2 and S4 for specific purposes stated in the figure legends. *B*, the presence or absence of transcription binding element found within 100 bp ± of PGC-1a peaks are shown. ERR, estrogen-related receptor; Gabpa, GA-binding protein-a; Nrf1, nuclear respiratory factor 1; PGC-1α, PPARγ coactivator 1α; YY1, Yin Yang 1.

Among transcription factors coactivated by PGC-1α, ERR, Nrf1, Gabpa, and YY1 are implicated in transcribing mitochondrial energetic genes (9). In contrast, PPAR targets are enriched in fatty acid metabolism (10). FoxO1 rather suppresses nucleus-encoded mitochondria genes (11). The Mef2 family plays a role in the differentiation, morphogenesis, and maintenance of several tissue types (12). Therefore, we primarily focused on ERR, Nrf1, Gabpa, and YY1 in this study. These transcription factors may recruit PGC-1α to TCA cycle gene promoters. To test this possibility, we first identified the transcription factor binding elements of ERR, Nrf1, Gabpa, and YY1 with bioinformatics analysis using transcription factors binding profiling. Their binding elements were found in TCA cycle gene promoters proximal to PGC-1α peaks (Figs. 3*A*, and S1, S2). Within ± 100 bp of PGC-1α peaks in the 13 promoters, we found 6 ERR, 5 Nrf1, 6 Gabpa, and 2 YY1 binding elements (Fig. 3*B*). Thus, these transcription factors may bind to the promoters *via* their binding elements and recruit PGC-1α.

### Responsible transcription factors transcribing TCA cycle genes

**Figure 4:**
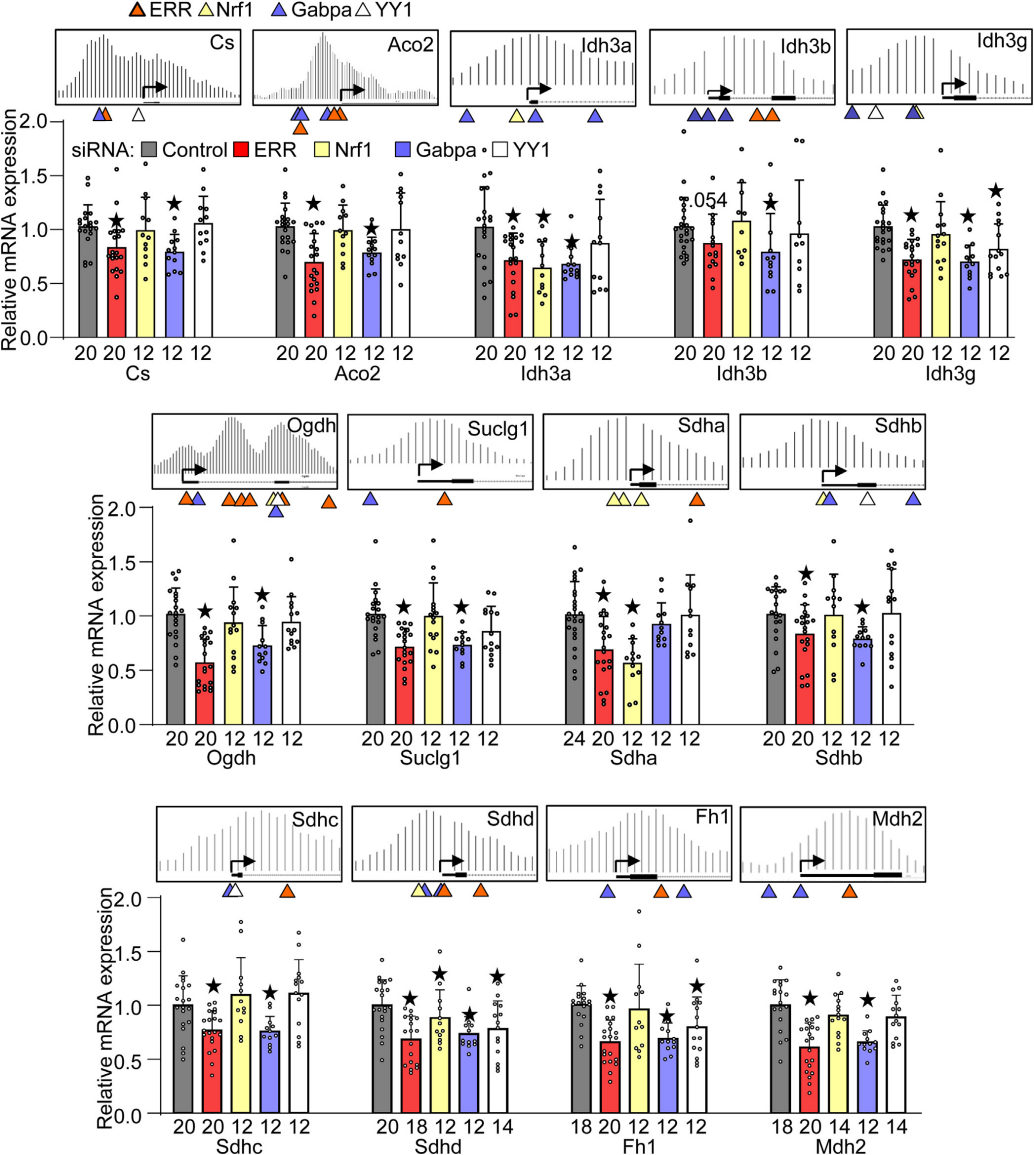
**Identification of transcription factor essential for transcription of TCA cycle enzymes.** Gene expressional analyses were performed in cultured cardiomyocytes with knockdown of indicated transcription factors with indicated n. ★*p* < 0.05 vs control siRNA. The ChIP-seq images are reused from Figure 3*A* to indicate the extent to which a transcription factor whose binding sequence is found proximal to the PPARγ coactivator 1α localization peak essentially transcribes the gene. ERR, estrogen-related receptor; Gabpa, GA-binding protein-a; Nrf1, nuclear respiratory factor 1; YY1, Yin Yang 1.

To investigate whether ERR, Gabpa, Nrf1, and YY1 are critical for the expression of individual TCA cycle genes, these transcription factors were knocked down with short interference RNA. Because ERR consists of three isoforms ERRα, ERRβ, and ERRγ, we first examined their copy number at mRNA levels. In both mouse hearts and cultured cardiomyocytes, ERRα was the most abundant (Fig. S3*A*). Therefore, we used siRNA sequence derived from ERRα, which has a one-nucleotide mismatch with ERRβ and ERRγ. As shown in Figure S3*B*, the siRNA primarily targeting ERRα successfully downregulated ERRα and ERRγ. We used two commercially available ERRβ antibodies, but we could not observe any ERRβ specific signal. Knockdown of Nrf1, Gabpa, and YY1 was also successful. Knockdown of these transcription factors did not significantly change the expression of the other transcription factors. To identify a critical transcription factor responsible for TCA cycle gene expression, mRNA levels of TCA cycle genes were examined. As shown in Fig. 4, the knockdown of the transcription factor whose binding element was found in the flanking region of PGC-1α peak(s) downregulated the downstream genes in many cases. As an example in Cs, the binding elements of ERR and Gabpa were found in the flanking region of the highest PGC-1α peak. Knockdown of ERR and Gabpa, but not that of Nrf1 or YY1, downregulated CS. This outcome was observed in all TCA cycle genes we tested. In contrast, we also observed two alternative outcomes as follows. (1) There is a transcription factor binding element near the PGC-1α peak, but knockdown of the transcription factor did not affect the expression of the gene. This includes YY1 knockdown in Ogdh, Sdhb, and Sdhc. These YY1 binding elements may not be functional. Alternatively, while YY1 binds to the element, it is not essential for PGC-1α recruitment due to compensation by the other transcription factors. (2) Although the transcription factor binding elements were not identified in the flanking region of PGC-1α peaks, knockdown of the transcription factor downregulated the gene. This example includes Gabpa knockdown in Idh3a and Suclg1 genes, ERR knockdown in Idh3g, Sdha, and Sdhb genes, and YY1 knockdown in Sdhd and Fh1 genes. Although our initial bioinformatics analysis could not identify their binding elements, relatively degenerated sequences of binding elements were found in the flanking region of PGC-1α binding peaks, including Gabpa in Idh3a, ERR in Idh3g, and YY1 in Sdhd and Fh1 promoters (Fig. S2). These binding elements may be functional to bind the transcription factors and recruit PGC-1α. In summary, among the four transcription factors, the knockdown of ERR and Gabpa downregulated 12 genes, whereas that of Nrf1 and YY1 did three genes. These results suggest that PGC-1α facilitates ERR, Gabpa, Nrf1, and YY1 to transcribe TCA cycle genes in a gene-specific manner. In addition, ERR and Gabpa play a major role in transcribing TCA cycle genes possibly through PGC-1α recruitment.

### Conservation of the transcription factor binding elements in the promoters

**Figure 5:**
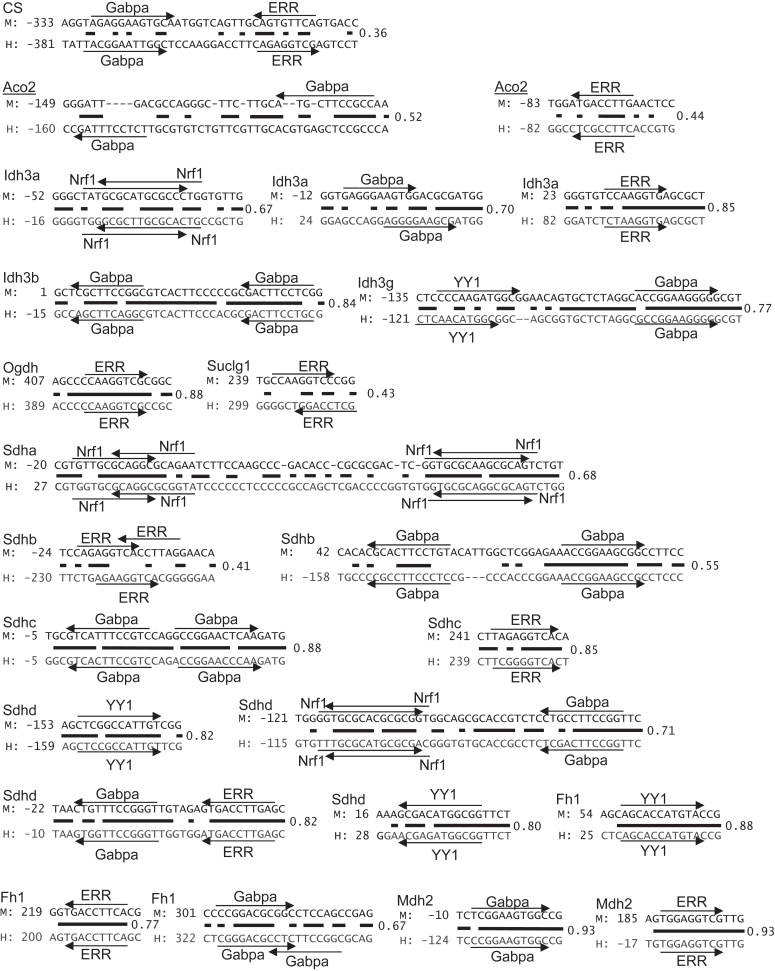
**The critical transcription factor binding elements are conserved between mice and humans.** Homology search in comparison between mice and human genomes was performed with the selected binding elements. M and H indicate mice and humans, respectively. The number indicates the position from the transcription start site. The back bar indicates the identical nucleotide sequence. The homology (ratio of identical nucleotides relative to total number of nucleotides) in the genomic regions is also indicated. ERR, estrogen-related receptor; Gabpa, GA-binding protein-a; Nrf1, nuclear respiratory factor 1; YY1, Yin Yang 1.

Transcription factors recruiting PGC-1α and their binding elements were raised by ChIP-seq, bioinformatics, and gene expressional analyses in mice (Fig. 4). PGC-1α is evolutionally conserved among vertebrates (13). To investigate whether and how the transcription factor binding elements are evolutionally conserved among vertebrates, we examined the promoter sequences of TCA cycle genes in mice, chicken, and zebrafish. We noticed that the promoter sequences did not show significant homology in these species. As examples with Cs, Aco2, and Idh3a promoters, transcription factor binding profiling showed different patterns and sequences in these species (Fig. S4). To investigate the conservation in mammals, a homology search was performed in mice and humans. As shown in Figure 5, these transcription factor binding elements were well conserved in the promoters, with some exceptions. The exceptions include the binding element of ERR in Ogdh and Sdha promoters and that of Gabpa in Sdha promoters. These binding elements in mice were not found in human promoters. In the case that the binding elements are conserved, we noticed two patterns of conservation. First, the binding element and its proximal region exhibited high homology between mice and humans, whose examples include ERR binding element in (1) Idh3a, Ogdh, Sdhc, Sdhd, Fh1, and Mdh2 promoters (2), Gabpa binding elements in Idh3b, Idh3g, Sdhc, Sdhd, and Mdh2 promoters, and (3) YY1 binding element in Sdhd and Fh1 promoters. Second, while promoter sequences did not necessarily exhibit high homology, identical transcription factor binding elements were still found in the corresponding region of the promoters. The examples include ERR binding elements in Cs and Suclg1 promoters. These binding elements were found in the corresponding region of the promoter, but their topology was the opposite. These results suggest that critical transcription factor binding elements are well conserved between mice and humans.

### Verification of the transcription factor binding elements in the promoters

**Figure 6:**
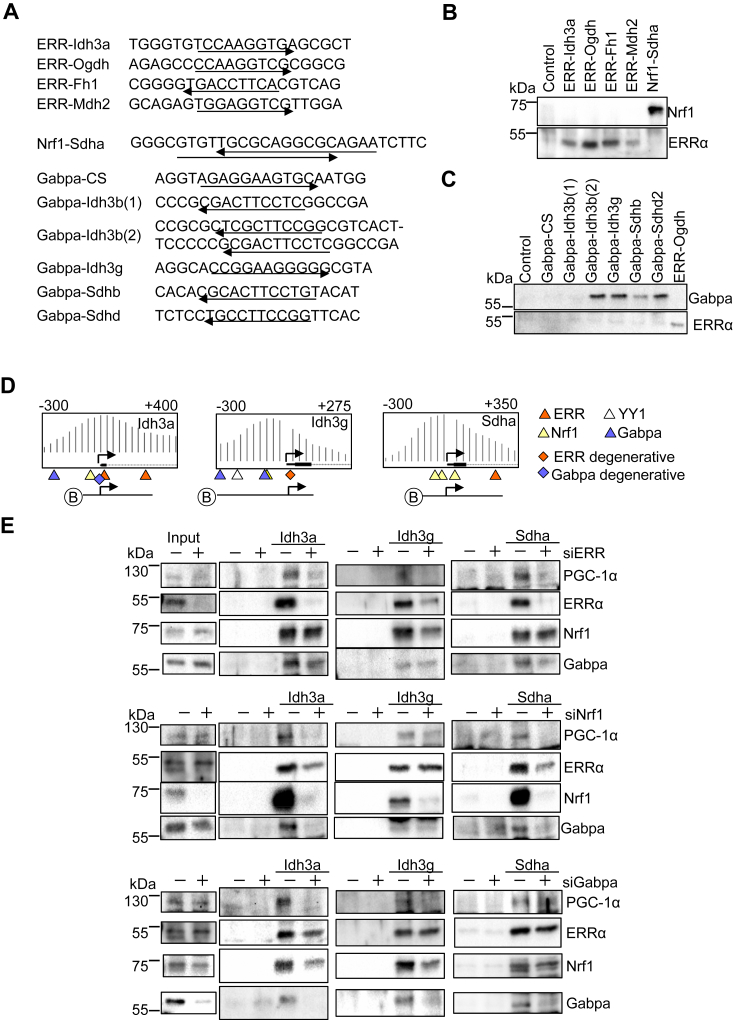
**The binding of ERR, Nrf1, Gabpa and YY1 to the TCA cycle gene promoters.***A*, biotin-labeled DNA used by this study. *B* and *C*, the binding of ERRα, Nrf1, and Gabpa to the indicated biotin-labeled DNA with nuclear extract from cultured cardiomyocytes. *D*, biotin-labeled DNA comprising indicated endogenous promoter sequences. The binding elements of the transcription factors are shown. The ChIP-seq images are reused from Figure 3*A* to indicate the promoter region with possible transcription factor binding elements used for DNA binding assays. *E*, the DNA binding assays were performed with indicated biotin-labeled DNA and nuclear extract from cultured cardiomyocytes with knockdown of indicated transcription factors. The results were verified with at least three independent experiments. ERR, estrogen-related receptor; Gabpa, GA-binding protein-a; Nrf1, nuclear respiratory factor 1; PGC-1α, PPARγ coactivator 1α; YY1, Yin Yang 1.

Because the possible transcription factor binding elements raised by the bioinformatics analyses (Fig. 3) exhibited a redundancy from their consensus binding sequences, DNA binding assays were performed with (1) ERR binding element in Idh3a, Ogdh, Fh1, and Mdh2 (2), Nrf1 binding element in Sdha, and (3) Gabpa binding element in Cs, Idh3b, Idh3g, Sdhb, and Sdhd promoters (Fig. 6*A*). Biotin-labeled double-stranded DNA comprising the endogenous binding elements was incubated with cardiomyocyte nuclear extracts. The transcription factor bound to the DNA was detected by Western blot analyses. ERRα bound to ERR binding elements derived from Idh3a, Ogdh, Fh1, and Mdh2 promoters, but not to the Nrf1 binding element derived from the Sdha promoter, whereas Nrf1 bound to the Nrf1 binding element in Sdha promoter, but not the ERR binding elements (Fig. 6*B*). As shown in Figure 6*C*, Gabpa bound to Gabpa binding elements derived from Idh3g, Sdhb, and Sdhd, but not CS. Significant Gabpa binding was not observed with a single Gabpa binding element (Gabpa-Idh3b (1)) in Idh3b promoter, whereas the binding was observed to that containing two binding elements (Gabpa-Idh3b (2)). To test the extent to which ERR, Nrf1, and Gabpa mediate recruitment of PGC-1α to the promoters, DNA binding assays were performed with biotin-labeled DNA comprising endogenous promoter sequences of Idh3a, Idh3g, and Sdha where PGC-1α localizes to and essential transcription factors’ binding elements are encoded (Fig. 6*D*). As shown in Figure 6*E*, knockdown of ERR, Nrf1, and Gabpa inhibited PGC-1α recruitment to Idh3a promoter, which was consistent with downregulation of Idh3a due to knockdown of ERR, Nrf1, and Gabpa (Fig. 4). Knockdown of ERR and Gabpa, but not that of Nrf1, inhibited PGC-1α recruitment to Idh3g promoter, which was consistent with downregulation of Idh3g due to knockdown of ERR and Gabpa, but not Nrf1 (Fig. 4). Knockdown of ERR and Nrf1, but not that of Gabpa, inhibited PGC-1α recruitment to Sdha promoter, which was consistent with downregulation of Sdha due to knockdown of ERR and Nrf1, but not Gabpa (Fig. 4). We also found that knockdown of a transcription factor inhibits the promoter binding of the other transcription factors in some cases. For instance, the knockdown of ERR partly inhibited the binding of Nrf1 to the Idh3g promoter and that of Gabpa to the Sdha promoter. Knockdown of Nrf1 inhibited the binding of Gabpa to Idh3a and Sdha promoters and the binding of ERRα to Sdha promoters. Knockdown of Gabpa inhibited the binding of Nrf1 to Idh3a and Idh3g promoter. These results suggest that these transcription factors mediate PGC-1α recruitment to a gene promoter in a context-specific manner. In addition, a transcription factor promotes the promoter binding of the other transcription factors in some cases.

### Dysregulation of the transcriptional regulatory mechanism in the failing heart

**Figure 7:**
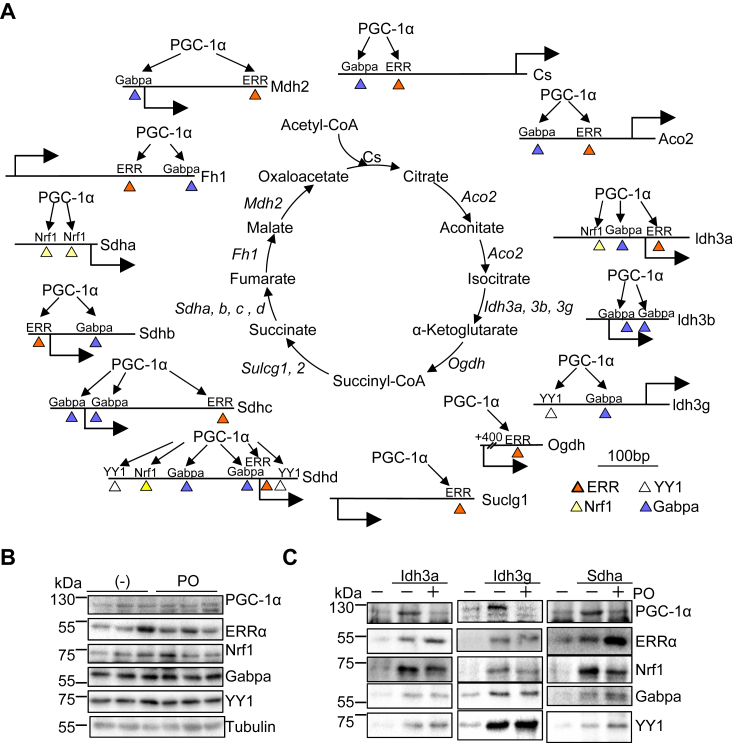
**Transcriptional mechanism for TCA cycle genes.***A*, Schematic representation for the basal transcriptional mechanism for TCA cycle genes. *B*, the expression levels of indicated proteins in the PO model and sham-operated LV as a control. *C*, recruitment of PGC-1α, but not ERRα, Nrf1, Gabpa, or YY1, is inhibited in the PO model. DNA binding assays were performed with nuclear extract of the LV subjected to 4 weeks of PO. The most representative images are shown with 4 to 6 independent experiments. ERR, estrogen-related receptor; Gabpa, GA-binding protein-a; Nrf1, nuclear respiratory factor 1; PGC-1α, PPARγ coactivator 1α; YY1, Yin Yang 1.

PGC-1α-mediated transcriptional regulatory mechanisms for TCA cycle genes in the healthy heart shown by this study are summarized in Figure 7*A*. PGC-1α transcribes TCA cycle genes *via* ERR, Gabpa, Nrf1, and YY1 in a promoter context-specific manner. To test the extent to which the basal transcriptional mechanism provided by this study is useful to clarify how TCA cycle genes are dysregulated in stress conditions, a DNA binding assay was performed with Idh3a, Idh3g, and Sdha promoters with heart lysate prepared from PO-induced heart failure model. Protein levels of PGC-1α, ERRα, Nrf1, Gabpa, and YY1 were not significantly changed in PO-induced heart failure model (Fig. 7*B*). As shown in Figure 7*C*, the recruitment of PGC-1α to Idh3a, Idh3g, and Sdha promoters was significantly inhibited in the failing heart. The binding of the four transcription factors was observed to some extent even if the transcription factor is not essential for the transcription of the target gene. The binding of Nrf1 to the promoters was slightly inhibited in the PO model. The binding of ERRα to Idh3a and Sdha, but not Idh3g, promoters was enhanced. The binding of Gabpa and YY1 to the promoters was not significantly changed. Thus, the downregulation of Idh3a, Idh3g, and Sdha under PO conditions is possibly due to inhibition of PGC-1α recruitment. Whether changes in ERR and Nrf1 recruitment inhibit PGC-1α recruitment needs to be carefully determined. Thus, the transcriptional regulatory mechanism provided by this study could be useful to clarify how these genes are dysregulated in disease or stress conditions.

## Discussion

This study aimed to answer a fundamental question of how TCA cycle genes are transcribed in basal conditions. There are many transcription factors that possibly transcribe mitochondrial energetic genes (9). However, how individual genes are transcribed remains unknown in many cases. Here, we took advantage of the molecular property of PGC-1α that essentially coactivates many transcription factors. Instead of performing ChIP-seq for some possible transcription factors, peaks of PGC-1α localization in the promoters allowed the identification of responsible transcription factors. This study shows that 13 out of 14 TCA cycle genes are direct targets of PGC-1α. This study also elucidates transcription factors and their binding elements that mediate PGC-1α recruitment (Figs. 7*A* and S5).

### Metabolic disturbance in heart failure

Downregulation of mitochondrial energetic genes is a hallmark of heart failure, which is generally thought to exacerbate the pathology due to insufficient energy production (8). This study showed that many TCA cycle genes were also downregulated in the PO-induced heart failure model (Fig. 1*B*). The basal transcriptional mechanism provided by this study could be useful to clarify how TCA cycle genes are dysregulated in pathological conditions such as heart failure. We clarified that the recruitment of PGC-1α to Idh3a, Idh3g, and Sdha promoters was impaired in the PO model with DNA binding assays. Consistently, ChIP assays demonstrate that recruitment of PGC-1α to metabolic gene promoters including Idh3a and Sdha promoters is inhibited in the PO model (5). How PGC-1α recruitment is inhibited in the failing heart is currently unknown. A posttranslational modification of PGC-1α or coupling transcription factors may be responsible mechanisms that inhibit its binding. Further study is needed to clarify the mechanism.

### PGC-1α biology

Despite the pathophysiological significance of PGC-1α, how the loss of PGC-1α dysregulates metabolites is not fully understood. A significant downregulation of succinate is observed in PGC-1αcKO mice (14). This study showed similar trends, partly supporting their conclusion that the TCA cycle is suppressed in PGC-1αcKO mice. A significant accumulation of citrate may indicate the same (Fig. 3*D*). Notably, however, a decrease in citrate and malate and an increase in succinate have been reported in the PO-induced heart failure model (15). Although PGC-1α inactivation is proposed as a cause of metabolic disturbance in the failing heart (16), loss of PGC-1α alone may not explain the changes in the metabolites. Besides the metabolites, how PGC-1α functions is not fully understood. Besides direct transcriptional activation *via* promoter localization, it is also proposed that PGC-1α induces critical transcription factors such as ERRα, Nrf1, and Gabpa, thereby indirectly inducing metabolic genes (17, 18). Whether PGC-1α potentiates transcription mainly through a direct or indirect manner has not been clearly demonstrated. This study showed that PGC-1α localized 13 among 14 TCA cycle gene promoters, suggesting that PGC-1α potentiates transcription through direct promoter localization of individual metabolic genes. Notably, there are only a limited number of PGC-1α direct target genes identified. Although many metabolic genes are downregulated in PGC-1α knockout mice (19), whether those genes are direct or indirect PGC-1α targets cannot be distinguished. Although promoter localization of PGC-1α is shown in several genes such as FGF21, Ehhadh, Acadm, and Idh3b (20, 21), this does not necessarily mean that PGC-1α is essential in the transcription of these genes. This study showed that 13 TCA cycle genes are PGC-1α direct target genes. Despite the general agreement that PGC-1α broadly induces metabolic genes through the activation of interacting transcription factors including ERR, Nrf1, Gabpa, and YY1, a responsible transcription factor mediating PGC-1α function has not been defined in most of the target genes. Here, we show that ERR and Gabpa play a major role in the transcription of TCA cycle genes compared to Nrf1 and YY1. Whether or not ERR and Gabpa play a major role in the other classes of metabolic genes such as glycolysis, fatty acid metabolism, and electron transport chain is an important consideration. PPARs mediate PGC-1α-induced transcription of genes involved in fatty acid metabolism (10), whereas Foxo1-mediates that of gluconeogenesis (22). It is likely that PGC-1α facilitates a specific class of transcription factors to transcribe a specific class of metabolic genes, rather than that PGC-1α facilitates ERR and Gabpa regardless of the target genes. Further studies are needed to address this issue.

### Evolutional conservation of the basic transcriptional regulatory mechanism

Nucleus-encoded mitochondrial genes are well conserved among mammals. However, compared to the actual coding sequences of the genes, the promoter sequences are not necessarily highly conserved. Thus, although it is generally thought that the transcriptional regulatory mechanism is evolutionally conserved, this concept has not been experimentally verified in many genes. This study shows that the binding element of critical transcription factors transcribing the TCA cycle genes that are found proximal to the PGC-1α localization peaks are well conserved between mice and humans, whereas the actual DNA sequence of the element and its proximal genomic region did not necessarily show high homology (Fig. 5). Thus, it is likely that PGC-1α and the coupling transcription factors also potentiates transcription of TCA cycle genes not only in mice but also humans.

### Synergetic DNA binding of the transcription factors

We observed that the knockdown of a transcription factor occasionally inhibited the binding of other transcription factors to the promoter (Fig. 6*E*). For instance, knockdown of Gabpa partly inhibited the binding of Nrf1 to Idh3a and Shda promoters. Among four transcription factors, particularly, the promoter binding of Gabpa often depends on the other transcription factors. Knockdown of ERR inhibited the binding of Gabpa to Idh3a and Shda promoters. Knockdown of Nrf1 inhibited the binding of Gabpa to Idh3a, Idh3g, and Shda promoters. Thus, although the binding of Gabpa to a Gabpa binding element in CS and Idh3b promoters was not detected with a 20 bp of double-stranded oligonucleotide context (Fig. 6*C*), Gabpa may bind to the element in the presence of the other transcription factors with a promoter context. The mechanism responsible for synergetic DNA binding is currently unknown. Transcriptional coactivators including PGC-1α and general transcription factors may bridge the transcription factors to support their promoter binding by forming a loop structure in the promoter.

### Limitations of this study

There are four limitations to this study. First, the basal transcriptional mechanism of Suclg2 remains unknown because it is unlikely a PGC-1α target gene. Second, this study was performed only with the use of mice heart and cultured cardiomyocytes. The extent to which the basic transcriptional mechanism is identical for other tissues is currently unknown. Third, the role of the other transcription factors coactivated by PGC-1α is currently unknown. This study verified a transcription factor that essentially transcribes individual TCA cycle genes among the four transcription factors we tested. Besides the four transcription factors, PGC-1α coactivates other transcription factors such as FoxO1, Mef2C, HNF4, and PPARs (23). Among them, PPAR is a well-established transcription factor coactivated by PGC-1α. Transcription factor binding profiling raises PPAR binding element proximal to PGC-1α localization peaks in Cs and Sdhb (data not shown). The extent to which PPAR and the other transcription factors essentially transcribe TCA cycle genes should be carefully determined. Fourth, our metabolome analyses detected only two metabolites in the TCA cycle. The limited number of detected metabolites is a reflection of the rapid turnover and low abundance of certain TCA cycle intermediates, which can be below the limit of detection (LOD) with current analytical methods. Moreover, the interplay between the TCA cycle and other metabolic pathways in the heart, along with sample matrix effects, can affect the sensitivity and specificity of metabolite detection.

## Conclusion

In summary, this study shows a transcriptional regulatory mechanism for TCA cycle genes in the normal heart, which is an important prerequisite to elucidate how TCA cycle genes are dysregulated in pathological conditions such as heart failure. In general, a transcription factor whose binding element is found proximal to the peaks of PGC-1α localization essentially mediates the transcription. Among four transcription factors we examined, ERR and Gabpa play a major role in the transcription of TCA cycle genes. There is a synergetic promoter binding of the distinct transcription factors to TCA cycle gene promoters *via* an unknown mechanism. The knowledge provided by this study provides a better understanding of PGC-1α biology and the fundamental mechanism for bioenergetics.

## Experimental procedure

### Animal experiments

Cardiac-specific PGC-1α knockout mice have been described previously (6). A pressure overload-induced heart failure model was performed with transverse aortic constriction for 2 weeks (6). Fasting was performed with free access to water for 48 h. All procedures involving animals were performed in accordance with protocols approved by the Institutional Animal Care and Use Committee at Rutgers New Jersey Medical School.

### Primary cultures of neonatal rat ventricular myocytes

Primary cultures of ventricular cardiomyocytes were prepared from 1-day-old Crl:(WI) BR-Wistar rats (Harlan). A discontinuous Percoll gradient was employed to isolate a cardiomyocyte-enriched fraction. Cardiomyocytes can be validated by observation with a microscope because of beating.

### Mitochondrial ATP production

Mitochondria were freshly isolated from the ventricle using a mitochondria isolation kit (Sigma, MITOISO1), which relies on differential centrifugation. Approximately 100 μl of mitochondrial fraction was generated from 50 mg of heart tissue. Mitochondrial ATP production was measured by bioluminescent reason with luciferase. The mitochondrial fraction (1 μl) was incubated with 25 μl ATP assay mix (Sigma FL-AA) 25 μl substrate buffer [5 mM Hepes pH 7.9, 210 mM Mannitol, 70 mM Sucrose, 10 mM Pyruvate, 10 mM Malate, 4 mM K_2_HPO_4_]. After 5 min of incubation at room temperature, the reaction was started by the addition of 1 μl 12.5 mM ADP. The luminescence was measured by a luminometer for 30 s.

### Immunoblot

The heart lysate was prepared with a lysis buffer (50 mM Tris pH7.6, 150 mM NaCl, 1% Triton-X100, 50 mM NaF, 10 mM EDTA, 10 mM sodium butyrate, 0.1 mM DTT, and protease inhibitor cocktail (Sigma)). Antibodies used for Western blot analyses were Gabpa (Novus 32105), NRF1 (Cell Signaling Technology D9K6P), ERRα (Cell Signaling Technology E1G1J), ESRRγ (Sigma AV45602), YY1 (Cell Signal Technology D5D9Z), PGC-1α (Millipore Ab3242 and Calbiochem ST1240) and tubulin (Sigma T6199). Besides tubulin and PGC-1α, the specificity of the antibodies was validated with immunoblot followed by knockdown with siRNA (Fig. S3). The specificity of PGC-1α antibody was verified with immunoblot followed by knockdown with siRNA and by overexpression with adenovirus vector (5). Tubulin antibody was validated with immunoblot showing a strong signal at the expected molecular weight (50 KDa).

### Quantitative RT-PCR

Total RNA was prepared from left ventricles using the RNeasy Fibrous Tissue Mini Kit (Qiagen) or from cultured cardiomyocytes using Trizol (Invitrogen). cDNA was then generated using M-MLV Reverse transcriptase (Promega). Real-time RT-PCR was performed using the Maxima SYBR Green qPCR master mix (Abclonal) with the following cycling conditions: 30 cycles of 94 °C for 5 s, 57 °C for 10 s, and 72 °C for 10 s. The mean value from wild mice or control cells was expressed as 1, unless otherwise indicated. RPS15 was used as an internal control. Relative RPS15 mRNA levels normalized by β-actin mRNA in PGC-1αcKO mice compared to wild type mice were 0.85 and *p* = 0.27. Primers used for investigating mouse genes are as follows: Cs - AGGCCCAAGTCCATGAGCACG and GGTGAGGGGAGAAGGGGTCA; Aco2 - CCAGGGCAGGACACATACCAG and AACTTGAGCCAGGGGCCGG; Idh3b - GGGAGGCTACAGCACCACAAC and ATATCTGAGGTTCGAACCTGTGGG; Suclg1 - GCTGGTATAACTGCTCCTCCTGG and TGTGCGGGGGACATGCTGACAA; Sdhb - GGTGGAACGGAGACAAGTACCTG and CTTGTAGGTCGCCATCATCTTCTTG; Madh2 - TATGCTGGAGCCCGCTTTGTCT and GTTAGGATGCTGCTGCTGCTGC; Idh3a - AACAAAACCCTTGCAATGGATGCACA and GGGTTAACAATGATAAATGATACAGTTCAC; ; Idh3g - CAAGGAACACAGGCAAAAGTATTGC and CTGGGGTATGCATATTTTCATTGTCCA; Ogdh - CACCATTGACCGTGCTAAGCCT and CGTCCAGGTCAAAGGCTGTGT; Sdhc - CAGTGGAAACAATGCCAGTTCTGT and GCTGCTCTTTATCTCCCTCTAGCT; Sdha - CGCACTGCAGACCATATATGGTG and GGGTGTGCTTCCTCCAGTGTTC; Sdhd - CTGTGGTGGACTACTCTCTGGCT and CATCGTGGTAATTGAAGTAGCAAAGCCC; Fh1 - GAGATGCTTCAGTGTCCTTCACAGA and CCCAGCATGTCCTTGGGTTTCAC; Suclg2 - GATAGCCAGGCCCAGCGCAT and GGCTTTGGTGATTCCGTTGGC; β-actin – AAGACCTCTATGCCAACACAGTGC and CACTTGCGGTGCACGATGGAG, and RPS15 - TTCGCAAGTTCACCTACC and GAGAACTCGCCCAGGTAGTGG.

### Proteome analysis

Label-free quantitative LC-MS/MS were performed with 3 month-old PGC-1α-cKO and control mice. The heart samples were diced into small pieces and washed with PBS. Proteins were extracted using lysis buffer (8M Urea, 100 mM TEAB, and 1X Protease Inhibitor cocktail (Sigma)). Total protein amount was measured by the BCA assay. 30 micrograms of proteins from each sample were digested with trypsin and the resulting peptides were desalted followed by LC-MS/MS analysis on Fusion Lumos MS instrument. The MS/MS spectra were searched against Uniprot mouse database using Sequest search engine on Proteome Discoverer platform (V2.4) and the relative quantification of the protein was calculated based on the LFQ.

### Metabolome analysis

LC-MS metabolomics were performed with 3-month-old PGC-1α-cKO and control mice using the method described previously (24). Hydrophilic Interaction Liquid Chromatography (HILIC) separation was performed on a Vanquish Horizon UHPLC system (Thermo Fisher Scientific) and an XBridge BEH Amide column (Waters) using a gradient of solvent A (95% water, 5% Acetonitrile) and solvent B (20% water, 80% Acetonitrile) with 20 mM acetic acid and 40 mM ammonium hydroxide at pH 9.4. For the mobile phase, ^2^H_4_-acetic acid replaced acetic acid in solvents A and B at the same concentration. Mass spectrometry analysis was performed on Thermo Q Exactive PLUS instrument. The levels of citrate were measured with EnzyChrom Citrate Assay kit (ECIT-100) according to the manufacturer’s instructions.

### ChIP-sequencing

Chromatin solution per immunoprecipitation was prepared by combining three mouse hearts (3 months old). ChIP-sequencing was performed with PGC-1α antibody (NOVUS, NBP1-04676) by Active Motif, Inc. The sequencing reads were aligned to the reference genome (mm10) using Bowtie2((25)). Peaks were called from the ChIP-seq data using MACS3((26)), and regions overlapping with the ENCODE blacklist, which are known to produce artefactual signals, were filtered out from the MACS3 output. The filtered peaks were then annotated using ChIPseeker in R((27)).The annotated peaks and motifs were visualized using IGB((28)).

### Bioinformatics analysis

ChIP-sequencing was performed on mice cardiomyocytes to locate DNA binding sites for target transcription factors: Estrogen-Related Receptor Alpha and Estrogen-Related Receptor Gamma (ERR), Yin Yang 1 (YY1), Nuclear Respiratory Factor 1 (Nrf1), and GA-Binding Protein Alpha Chain (Gabpa). Integrated Genome Browser (BioViz) was used to overlay ChIP-sequencing data and known mice genome. Signal spikes, indicating transcription factor binding, near promotor regions of TCA enzyme genes were found with their corresponding length and base pair number. TCA enzyme promotor region sequences, along with upstream and downstream margins, were obtained from Ensemble (EMBL-EBI). The obtained sequences were scanned using JASPAR to cross-reference known genome sequences of ERR, YY1, Nrf1, and Gabpa at an accuracy of 0.85. Identified transcription factors were then graphed at their corresponding promotor region.

### Knockdown with siRNA

Small interfering RNA (siRNA) for rat Gabpa (SI01514807), YY1 (SI03026527), Nrf1 (SI03074463) and control siRNA (1022076) were obtained from Qiagen. siRNA for ERR (ESRRA0B1G1, SIC0056938) was custom made for the target sequence: 5′-AGGGCAAAGTGCCCATGCACA-3`. The siRNA was transfected with Lipofectamine RNAiMax (Invitrogen). 4.5 μl of Lipofectamine RNAiMax was diluted with 125 μl OPTI-MEM. 2 μl of 20 μM siRNA was diluted with 125 μl OPTI-MEM. The diluted Lipofectamine RNAiMax and siRNA were then mixed and incubated at room temperature for 20 min. The mixture was added to cardiomyocytes plated on a 6-well plate with 2 ml DMEM/F12 medium. The cells were used after 48 h of transfection. The protocol for Gabpa KO was modified where the cells were used after 5 days of transfection.

### *In vitro* DNA binding assay

Cardiomyocytes were lysed with 300 μl of a nuclear lysis buffer [25 mM Hepes, pH7.9, 0.1 mM EDTA, 10 mM Sodium Butyrate, 10 mM NaF, 1 mM DTT, 1% NP-40, 420 mM KCl, 1xProteinase inhibitor cocktail]. The 100 μl lysate was diluted with 300 μl of a dilution buffer [10 mM Hepes, pH 7.9, 2.5 mM MgCl2, 5% Glycerol, 1 mM DTT, 0.1%NP-40]. The total 400 μl of diluted lysate was incubated with 0.3ul of 50uM biotin-labeled DNA and streptavidin-beads at 4 °C for 2 to 4 h with rotation. Biotin-labeled endogenous promoter sequences were also used. The protein-DNA complex was washed with 1 ml of a washing buffer [10 mM Hepes, pH 7.9, 2.5 mM MgCl2, 5% Glycerol, and 50 mM KCl 1 mM DTT, 0.1%NP-40] 3 times. The protein bound to the DNA was analyzed with Western blot analyses. Double-stranded DNA used for the pulldown assays was following. Biotin was conjugated to following forward oligonucleotides. Non-biotinylated reversed oligonucleotides were annealed with indicated oligonucleotides (5′-3′). ERR-Idh3a: TGGGTGTCCAAGGTGAGCGCT, ERR-Ogdh: AGAGCCCCAAGGTCGCGGCG, ERR-Fh1: CGGGGTGACCTTCACGTCAG, ERR-Mdh2: GCAGAGTGGAGGTCGTTGGA, Nrf1-Sdha: GGGCGTGTTGCGCAGGCGCAGAATCTTC, Gabpa-CS: AGGTAGAGGAAGTGCAATGG, Gabpa-Idh3b (1): CCCGCGACTTCCTCGGCCGA, CCGCGCTCGCTTCCGGCGTCACT TCCCCCGCGACTTCCTCGGCCGA, Gabpa-Idh3g: AGGCACCGGAAGGGGGCGTA, Gabpa-Sdhb: CACACGCACTTCCTGTACAT, and Gabpa-Sdhd: TCTCCTGCCTTCCGGTTCAC. Biotin-labeled promoters was prepared by PCR with a biotin-labeled primer as follows. Idh3a: CGAGAGACCCGAGCTAGGG and TGGGGACACCCGGAGCAGTAC, Idh3g: GACATGCGGAGCAGACGAG and CCAAGGACGGCAGAGGAGA, and Sdha: GTGTTGCCACCGCCGCGTT and TCCAGAATGCAACAGGCCGGTC.

### Statistical analysis

Statistical comparisons were made using the unpaired and two tailed Student’s *t* test. *p* < 0.05 was defined as statistically significant and is indicated by a filled asterisk. *p* > 0.05 is indicated by NS. All error bars represent SD.

## Data availability

All data provided by ChIP-seq, proteomics and metabolomics used for this study are available in figure share (https://figshare.com/articles/dataset/ChIP-seq_with_anti-PGC-1_antibody_and_omics_analyses_in_cardiac_specific_PGC-1_knockout_mice_related_to_TCA_cycle/26103028). All the other data provided by these experiments are available upon request to the corresponding author (okash@njms.rutgers.edu) unless any conflicts exist with the investigators.

## Supporting information

This article contains supporting information.

## Conflict of interest

The authors declare that they have no competing interests with the contents of this article.
