# Supplementary material for: A transcriptional regulatory mechanism of genes in the tricarboxylic acid cycle in the heart
Source: J Biol Chem. 2024 Aug 14;300(9):107677. doi: 10.1016/j.jbc.2024.107677 (PMC11415578; doi:10.1016/j.jbc.2024.107677)
Supplement: Supporting Figures [file mmc1.pdf]

Figure S1

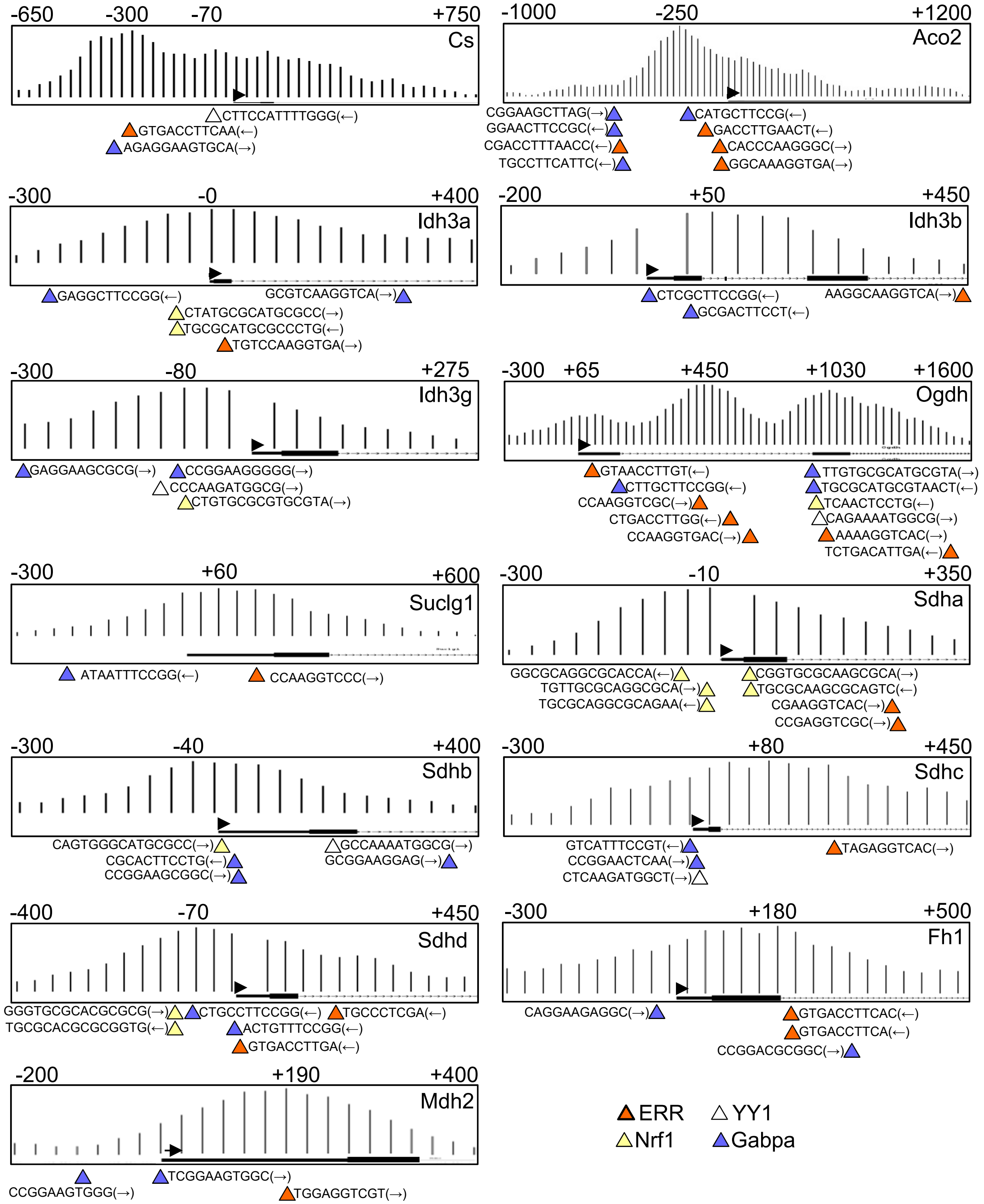


**Figure S1: Possible binding elements of ERR, YY1, Nrf1, and Gabpa in TCA cycle gene promoters.** Bioinformatics analysis with transcription factor binding profiling identifies possible binding elements of ERR, NRf1, YY1, and Gabpa in TCA cycle gene promoters. Distance (bp) from the TSS and the peak PGC-1α location relative to the TSS are shown on the top of the panels.

Figure S2

# Cs

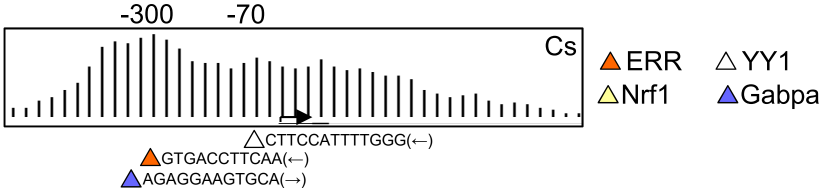


-500 TCTTCTCCACATAACGGGGTCAGTATCTGTAATTTCAAGATTTCTTCTGTTCACAAATGACCATCAAGCAACTAACTCTGGCATCATAGCGGGCTTTCTG

-400 CGTGATGTCACCTCCACGGCCATGAAGTCATTAACCAAAGACATTCTTTAAGAAAGCATGCCAAGCAGGTAGAGGAAGTGCAATGGTCAGTTGCAGTGTT
Gabpa

-300 CAGTGACCTTCAAACGTCGCGTGCTGGAGCTTTATCTTCAGCATTCTCCGGAGAACCCGAAATGTGTTAAGGGCAAAGGGAAAAGCCAAAGAAGGAAGTT
ERR

-200 AACTGAGTTCTCAGGCACATCCTTAGACACCTACAATCCCTTTCCCACCCACTCCTAATTAGCCTAATCCGTACGGTCCAATCCTCCCCGCGATAACATC

-100 TGTCAAAGGCTCAGATCCCGCCCCTCTTCCATTTTGGGCCAATGGTCGAGGCCGGGGTGGGTCGCCGGAGGGGGGCGGGCTCAAGTTTCGGGCAGAGGGC
YY1

1 GGGGCCTCGCCGAGGACCCTGGGCTGGGCGCCTGTGCCGGTTTGTCTACCCTTCCCCTCAGCCGCCTCCTTTCAACCTTGTCCGCCGGTTGGCGCGGTCT

101 CCATCACAGCGGCGACCGCTCCTGTTGCAGCTGTAGCTCTCTCCCTTCGGTCCCTTCCCGCCAGGTCCCGGAGTTTCGCCCGCCATGGCTCTACTCACTG

# Aco2

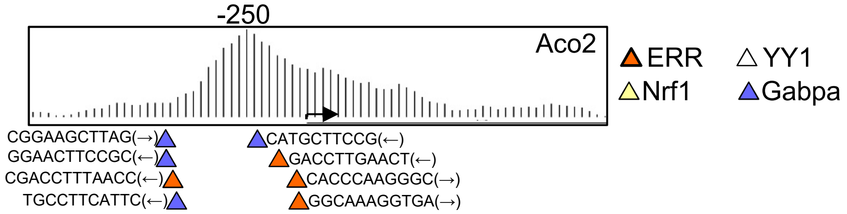


-500 TCACCCACACCAGCCTGCTTGCGGCAGAAAATCAAATCTGGATGATGTTTAGCCATAACTCTTTTTCCAAGCCGGCGGCCGGAAGCTTAGGGAACTTCCG
Gabpa Gabpa

Gabpa
-400 CCCGACCTTTAACCTACGGCGTGCTTACTTCCGTTCCTCCGTGATTGGCCATTAAGCTGTCCGTCAGCCTTTAGCCTCGGGGGGTGGGGGGGGGCACCAA
ERR

-300 AACCGGCTAGATCTCGCGAGAGTTTCCTCGCTAGGTCCTTGCAGGTGCACCAAAGGTTTTTGAATAAAATTCTCTTTACTTCCAGATTTGAACTCATAAA

-200 CTGAAGTTTGTTCTTGGGTAAAAATTCTACATATGTTGCATTCTGTGTTGGGGATTGACGCCAGGGCTTCTTGCATGCTTCCGCCAAGTATGTTGATTTG
Gabpa

-100 GGGATATTCTCTTTCCTGGATGACCTTGAACTCCTGCCTCCCCTCCAGCTTCCACACCCAAGGGCAAAGGTGACAAGGTTGCGCCGAACGCCGAGTTGGC
ERR ERR ERR

1 AGTTTCCCCGCATCTTTGCTTTCCACGCCGGGTTTCCGTCATGTAAGCCCCGCCCACTGTGCTATCTGCACTCTTGGCTGCGGGAGGAGGCCCCACCCTC

Figure S2

# Idh3a

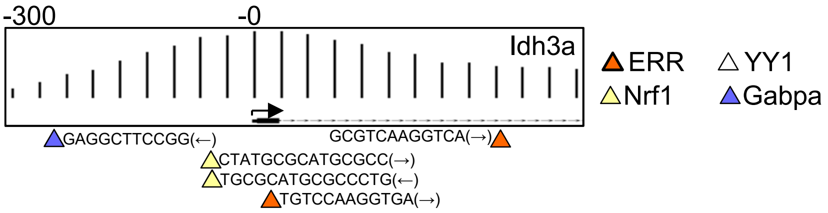


-300 AGGTCGGAAACAAAAAACTCATCCAAACAGCTTTTCAATGAATGAAGCGGGAGGAAGGGGAGGAGGCTTCCGGGTCGCAGCTGGGCCCGCTCCGCGCGTC
Gabpa

-200 ATCAAAAGATGAAGGCGGTGATTGGTCAGCTAGCGGCAGCTGTGCAGCGGGGTCCGGAGCGGGGCGGTGAGCGATCGAGAGACCCGAGCTAGGGGCGGGG

Nrf1
-100 CCACACGGAGCCTCGGGGCGGGGCCGTGAGGGGCGGGCTTGGGAAGAGGGCTATGCGCATGCGCCCTGGTGTTGCCGCTGTCGCCGCGGTGAGGGAAGTG
Nrf1 Gabpa

1 GACGCGATGGCCGGGTCCGCGTGGGTGTCCAAGGTGAGCGCTACAGGGCAGCAAGCTCGAGGGCGAGCAGGGAGAGTAGGCTGTGATCCCGCCTGATGGA
ERR

101 TGGCGGAGGCGAGCGCGCGGAGGCGGCTCATGGCCCGCGGGCAGTGTTTCGGGGAATGTGGCCGGCACGCCGTGCGTGGCTAGCCCTGAGCCGGGACAGC

201 CGCGCCGGGCGGGGACGGCGTCAAGGTCAAGGCGGCCAGTACTGCTCCGGGTGTCCCCACCAGTGTCCTCCAGGATAGGTGCACAGGCCTCCCCCGGAGC
ERR

# Idh3b

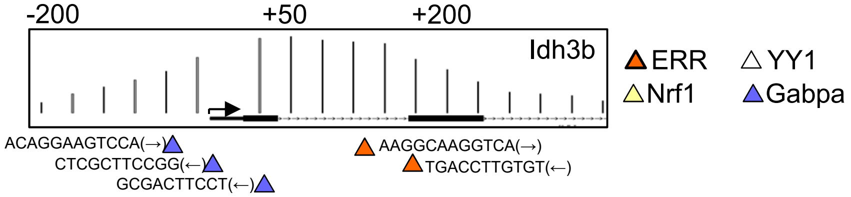


1 GTTGTTTTGAATTTTCAAGAGAAATTAAATCTGACGAGTGAAAACAAAGCAAACAGACAAACTGGTTGTGTGTCCTGTGACCCTACATTACCAAACAGAA

101 GGAGAGAACTCAAGTGAATCCGTACACACCGCCGATTGGTACGGGAGAAAGAACCGCCAAGCTCGCCAGGGCTGACAGGAAGTCCAGCTCCCACGACCGC
Gabpa

201 GCTCGCTTCCGGCGTCACTTCCCCCGCGACTTCCTCGGCCGAACATGGCAGCGCTGAGCAATGTCCGCTGGCTGACCCGAGTGAGGCCCGGGACGACCTG
Gabpa Gabpa

301 AGCGGAGGGTGGGCGGGAGAACAGAGGGTTTGTCCGCGGTTTCCAAAAGAAGGCAAGGTCATCGCGGTGTAAGGGGCCGCGGTGTCCCCGATCTGACCTT
ERR ERR

401 GTGTCTGTCGCAGGCGGTGCTCGCCGCTCGGAACTCCGGGGCATGGAGAGGTCTCGGAACATCTACGGCTCACGCCGCTTCCCAGAGCCAGGTACGCTTG

Figure S2

# Idh3g

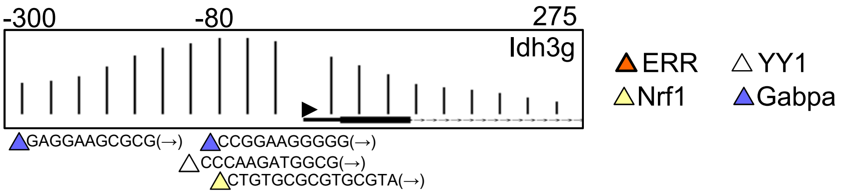


-300 GACATGCGGAGCAGACGAGGAAGCGCGACGCGTAAGCGGCCTCAGCACCCAATCGTGCGTCGGCGGGCTGGGTCGGGCATGGTGACGTCGCTGGCGGCAC

Gabpa

-200 TGACGTGTCCGCTTCCCTAGCCAGGGTCCCAACCGGTCGCACTTCCCTCCTTCTCAGCCTTCCCCTCCCCAAGATGGCGGAACAGTGCTCTAGGCACCGG

YY1

-100 AAGGGGGCGTAAGAACCGGTGTGACTGTGCGCGTGCGTAATGCTAGCGTTTTCTCAACCCATACCCCGGATTTCTCGCTGTCCCCAACTGTCGCGAGAAC

Gabpa Nrf1

1 GGTGCTTAATGTTTTGACCTGTAGAGGTCCTCACTTTTCGTCATGGCGCTGAAGGTGGCGATAGCTGCTGGCGGTGCTGCAAAGGCAATGCTCAAGCCAA

ERR

101 CTCTCCTCTGCCGTCCTTGGGAGGTGAGTGGTGAATGGGCGGTGGGACATTTGCAGGCTTACAGAGGGACTTAACACCCA

# Suclg1

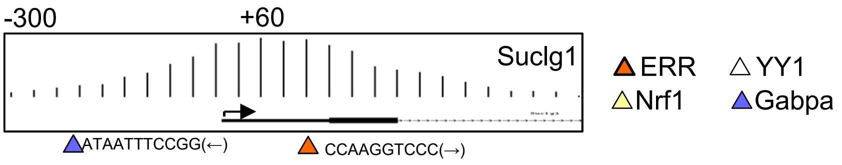


-300 TCAGGAAACTGTCAGCTAAGGGGCTTTTTAATTTTAAGTATTTGGTCACTTTTCCTTTTTTCTTTTGCCGGCCACTAGTGCGGATAATTTCCGGAACCGT

Gabpa

-200 ACAGGGAGGACAAGAGGAGTACAATCTTCTCTGTAACAGGTCAGAAAAAAACAAAGTGCAGGATCACGGTCTGGCACATAGCATTGCTGCCGGCGCCTCC

-100 CCTCAGCGTTTGTCACGGCTTTTCCTGGGCGCCCACGCCGGAGTTTAAATCGCTTCCAACCCCTTCCCTTTCAGCACGCTGCTTACTCGGAAAAAAAATA

1 CATTTCCCACAACACCACGCGCCGGCCGGGCCTCTCATGGGCTGTCCCACACATTGCCGTTATGTTTATGGTTACCCAAGCCCACATCTACTAAAGAACA

101 GAGCTATTGTTTGCTTTTCAAACGTCATCCTTCTTGTTCTTTTCCACTTGTAACGTTCACTGTTTGAGAAGAGACTCCGAGGAGCGTAAGATATTCAGGC

201 TACTCCTCTAGACACCTTTCTGCGCGAGCCTGAGCCCATGCCAAGGTCCCGGGGTCCTGAGAGTGTCATTGGGGGATGACCGCAACAGTAGTTGCTGCGG

ERR

Figure S2

# Ogdh

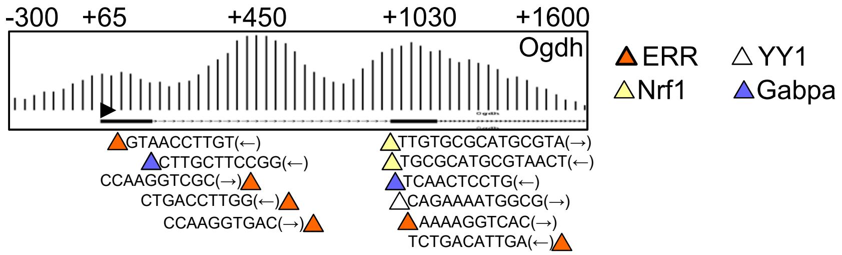


-300 AGTCGCAGCGCTCCTGCTTCGGCCCGCCAAACGCTTCAATTATCAGACGGCATCCCACGCCCTGAATGTACCAGGTTCTTAACAAGCTTCGGAAGCGTCT

-200 CCCGTGTAACTGCTAATGACAGCCGAAAGACAGTGAGCAACAGGCTGGCTTTGGCCAGATGCAAAGTTCTGCATTGGCGCGAAGCCCGAGCGAGCGACTG

-100 AAACCCAATTCTGTGACGTCACGTCACGCCCACAGCCTGTCTTGCAGGCCGCTCCTCTGGGGCCGGGCTACGCGTTGACGCCACACGCCGGGGCGGGGCT

1 GAGAGTTTGGAGCCCGGAGTGGTGTCGGCGCTTCACTCGGGTGGAGCTGAACGGGAGACAGGTAACCTTGTGCGGCAGACGGGCTCCGTCCAGGGCTGGG

ERR

101 AGGCTCGGCGCTTGACACCCGGAGCTCGCGGCCGAGGGTGAGGCCTATGCGTCCTTGCTTCCGGAGTGCCTGAGTGACGGGCGTGAAGCCGGACAGAGCC

Gabpa

201 GGGCTGGCGGAGGGCGGCGATGGTGGGCGGGGGACGAGACCTGACGGACAGACGTTATAGCCAATGGCGTGGCAGGGTCCTCCAGGCGGGCGGAGATTGG

301 CGGACGCGGACTTGCGCCGGGGGTCGGGGGGCCGAGCGCTATTGGCTCTGGCGGCGGAGCCGCGAAGGTGGCGGGCTAGGGAGTAGGTGGCCTGGCGGCC

401 CGGGAGAGCCCCAAGGTCGCGGCGTGTCACGTGGACCGGTCGCTCCCTCTGGCCCGCGCGCGTTCTTTTGTCCTGCGTGCGCTGACCCCAGCAGGCCGCC

ERR

501 GTCCTTGTGTTCGCCGTGCCCGGACACGGCGCTGACCTTGGTGCCTGCGGCAGCCCCACCTGCCGGTATGAGGTAAACCAGGCTGTCGGCCAAGGTGACA

ERR ERR

601 CTGGTTTCCCTAGAAACACCATCCACGCACCGCTTTCCTCGCGAGAGTGTGTGACAGATGTGAGTTATACCCAGAGTGACTGGGAAGCCTGCTACTGTGC

701 CCTCGCATGAGTTACCAGATCATGTTTCTTTTGCCCACCGAGCAGTGTGCCAGTCTCTGAAGTGACAGGTTCGGCAAGAATCCTCACGTGGATACCATGT

801 AAAGACAGGAGAGCGGAGTGTAGGGCATTTGAGTATCTACACGGTGAAGGAGCAGGGTGAGGTGTGGAAAAGGGATTAAAGGTAATGAGAAGTGACGGGC

Nrf1

901 TAGGTGTTGTGCGCATGCGTAACTCAACTTCCTGACTGTAAGATCAGAAAATGGCGGCGTTAAGCATCATTGGAGGGTGGAGTTTTAGATCCTTGACGTC

Nrf1 Gabpa YY1

1001 AAAAGGTCACTCAAGGAACACCTCTACAGGCCCAAGTGGGGTCAGTGGGGTCAACCTGTATTCGATGGAGGTCTCTACGTCCGTAAGTGTTATCTGTATA

ERR

1101 TAGTGAAGCTATTGCGAATGTGCAACTGGTCTTCATCACCCTAAGTAGAAGCGTGTAACTAAAAATAGAAGTAAAAATTTCCCCCAGCTTTTCTGTGGAT

1201 TTCATTGCCCTTACAGGCCCTACTTGTGTCTGTGTATCCTGTGCTCTGACATTGAGCAGAGGACACCGTTGGAGTTCCTCTGTTTGTCTGATCCTCTAGC

ERR

Figure S2

# Sdha

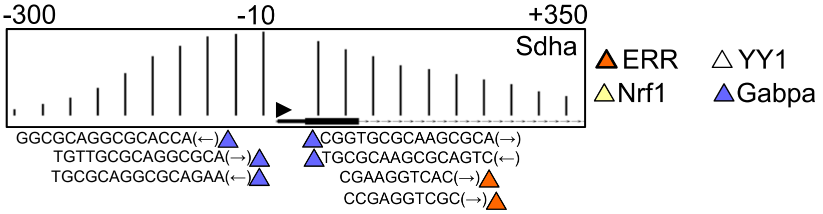


-300 CCCCCCCCCCCCATTAGTTGAGATTCAAGCGCCCAACAAAACGGGCTCTTATGGAGCTTGCGATCTCCGCCCCAGGACCCTACTTCCATCTCTCCAGATC

-200 ATCCGACCCTCTCTGTTTTGGCAAACAAGAACCACCCGAGAAGCACTTTAAAGACATTGGCACCTCACTTACCCCTGGAGCAGAGCCCATCAGCGTTGCT

Nrf1

-100 GCCACCCTACCTCAGACGTGTTGCCACCGCCGCGTTTACAGGGCGCAGGCGCACCACCACTCACGCGAGCCAGGAGGGGCGTGTTGCGCAGGCGCAGAAT

Nrf1 Nrf1

Nrf1

1 CTTCCAAGCCCGACACCCGCGCGACTCGGTGCGCAAGCGCAGTCTGTCGCTGGGGCTTGCGGGAAGGCAGACATGGCCGGGGTTGGCGCAGTTTCGAGGC

Nrf1

101 TTCTTCGCGGGCGGCGCTTGGCGTTAACTGGGGCGGTGAGTTAGTGCCACGAAATGGCAGGCGCTAGGAGTGCGCCACGATTCCACCCGAGTTGGCCTGA

201 GTCTGGGAAGGTGCAGTGGGCCGAAGCCGACTGCTTTCCGGCGAAGGTCACGACCCCGAGGTCGCGGGCGTGGAGTCTGGCCTTCCCCGTCTTGGTCTAG

ERR ERR

# Sdhb

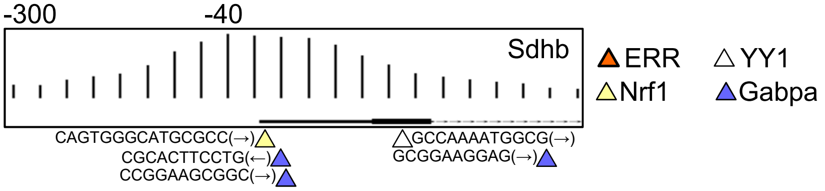


-300 ACTGTCTCCATATGCCGGGCATGCAACTTCTGTACCGACGTGACGTTCATCTTTTGATCACTTCAGTCACTTAACAAGTGTCCCAATCAGGTAGGGACTG

-200 ACAGGTTCTCTAGATTTAGCACAAAGCTGACCAGACAAGAGTCACAGGTGATTGACAGACACCACTGCAAGTAGAGAAGGGTTAAAAATCACAGCCACCA

-100 GCAGAATAGGAAAGCCCCGCCCTCCTGGCCTCTGCCCCGCCCCTCCACGCTACCCTTCCCTCTCCCGCCCCTCAGACTCCAGAGGTCACCTTAGGAACAT

1 AGGAAGCCCAGTGGGCATGCGCCCTACGGGCCGCTGCTGCACACACGCACTTCCTGTACATTGGCTCGGAGAAACCGGAAGCGGCCTTCCACTCGTTGGC

Nrf1 Gabpa Gabpa

101 GCTTAGGCGGCTAGCGGTCCTCAGGGTGAGAGGCCGGCTTCCCACCGCGGTGTTCGACGGGATGCCGGAGGGGAAGGGGTGGGGCTGACGTCAGGAGCCA

201 AAATGGCGGCGACGGTCGGGGTCTCCTTGAAGCGCGGCTTCCCGGCTGCCGTTCTCGGCAGAGTCGGCCTGCAGGTGAGCCCGGGAGCCGTGGTTCAGTG

YY1

301 GGGAGCCGTGCCCGTCACCGAGGCTGCCTGGGTAGAGAGGCCGCGGAGAGGCGGAAGGAGCCCGGGTCCAGGCCTCGGGGGAGGACTTAACCCGGCATCC

Gabpa

Figure S2

# Sdhc

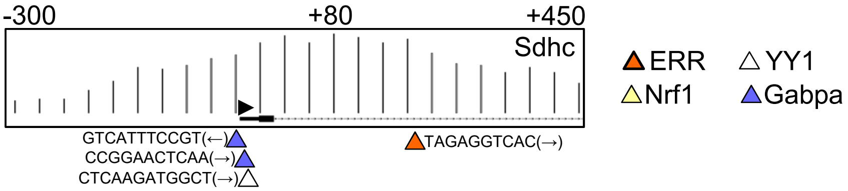


-300 GCAGGTAAACGCACTAACGGTAACCACTCGGCGATTTTGGCCAAAACTTTGACAAACGACGCTAGCTAGAAACTGGCGCGTGAGAGGAAAACCGAAGAAC

-200 TGAAAAGCAAAGCAAAACAAAAAACTAAAACCAAAAAAAACCAAAAAGAACCCAAAACAAAAAAAGTACCATCCACCAAGCCCGGAACACAAAGGAGGTT

-100 CCAGCCTAAAAATTCTGAGGTGTCATATGATAGTCCCCGCCTCCGCCCCGCCGGCTCCGCCCCCAAAGGCAGGGCCACACGGGGGGAGGAGCGATGCGTC

Gabpa (←)

Gabpa (→)

1 ATTTCCGTCCAGGCCGGAACTCAAGATGGCTGCGTTCTTGCTGAGGTGACTTTAGCCTGGGGCTAGGAGTCCACGCCCGCGACCGTTGGGGAACCTGAA

YY1 (→)

101 TTAGTCCCTGAGGGGGTGACAGCGGTGTGTGCCTTGCGGGGACAGGGAAAGGACCTGCGGGTGTGCAGGCCGCGCCCCTGGGATCTTAGCACCCCCTCTT

201 CTCCCCCGGCGGAGAGGCTCGGAAGGGCTTAGAGGTCACACTGTGTGTGGCCGAGTGGCGCGGCTGTCATTGTTTCTGCCTCGCCCACCTCTAAAGCCTC

ERR (→)

301 TTCGAAAACTGCTCTTGTGGCCCCAAAAAGCTGCTGTAACTTCGAGATCAGCGGGCCCCTAGGCCTGCAGTTTTGGGGGGACTTAGGGATTGTAGAGGGA

401 GGGGCTCCTTGGAGCACCTACTGGGTGCTTTAACTCCTGGCAATCCTGCTAGCAACCGGTGGAAACTGTCGGGCATCCTAATGCATCCCTCCCAAGCCAA

501 GGTCAATAATAGCATCCACAGTGGGTCTCTTAATGTTGCC

ERR (→)

Figure S2

# Sdhd

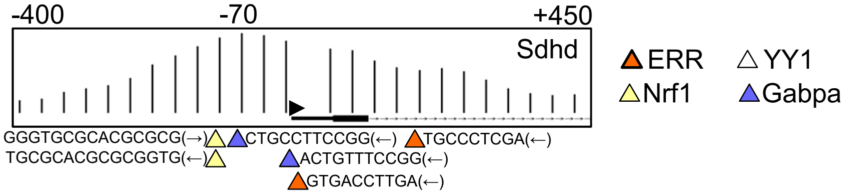


```
-400 ACCCTTTCCTCCCACGCAGCGCTTGCCTCCCGGGCACTGAGCACTGTAGATCACTGGGGCGGGAGGCGCAGCCCGGATCGAGTGGGGCCGCTGTGCCGCA

-300 GTGTCCAGAGCTCCGTGAGTGCCCGGGATCTGCGCCTCACCCTCCTTCCCCTGTTCCTTCCTCATCCTCGTACCTGCGCAGTGAATTGCGCCTTCTGCTG

                                                                                                    Nrf1
-200 TTCGGCGGCCACCAGGCGTTGTAACTCCGCTTCGTCCGCTTCACCAAGCTCGGCCATTGTCGGAGTCAGGCGCTTCGCTGGGGTGCGCACGCGCGGTGGC
                                                     YY1                                                 Nrf1

-100 AGCGCACCGTCTCCTGCCTTCCGGTTCACGCTTCAGGTTGCCTCACCGTTCTTTTACGCGGGGCGGGACTTACTACATAACTGTTTCCGGGTTGTAGAGT
                    Gabpa                                                                 Gabpa

   1 GACCTTGAGCCCTCGAAAGCGACATGGCGGTTCTCTTAAAGCTGGGCGTTCTCTGCAGTGGCCAAGGAGCTCGAGGTGAGGTGTCTTGGCCATCAAAGGT
       ERR                     YY1

 101 TCTTGCTGCAGGCTGAAGCAGGGGAGAGGAAGGAGTTAGAGCTCGCCCGCTGGGTGGTGGGACGTCTTGAGAGGAGGGAACTTGCTCTTGTGCCCTCGAG
                                                                                                      ERR

 201 GCACCACCTATGCGGACGCAACGTTGACCGCTATAGCCTAAAGGCCCAAGTGTTTACGTGCTGGCTGGTGTCCCCAGCTCGTACTTTCCGCTTCTGCACT

 301 GTGGTTGTGACCCCGGGCTAATATGAAAACAAACTCCTATTTGAGTGTGGATGGAAGGAGTCCGTTTGATAATTTGTTCTGAGTGGCTTGCTGTGTAGTG

 401 CTGTCTTCCTCGATCTAGCCATGTAGACAAGACTAACCTAGAGCTTGCCGGGAGCCCCGTGACTGCTCCCGAGGGCTGCCATAAAAACCGTGCACCCTCA

 501 CTCAGGGCTTCGATAATCATTTTAACCAGGCATTTTACCTATACCAGGCGCCTAAGAGGC
```

Figure S2

# Fh1

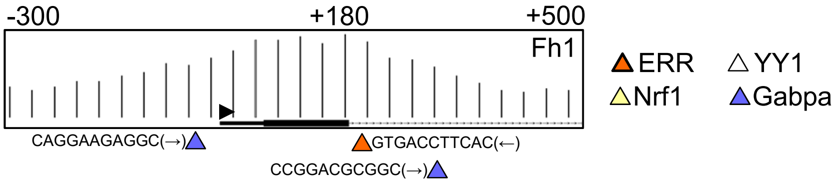


-300 ATTAACTTGATAACATGTTCTTTCTAGGCTGTCTCACGGACCTAGTACTACAGGCATCTTCAGCCAGTTTCCTGCCTCCTAGCAGCCACATTTGAAGTAT

-200 TGTCGGAAGATCTGACCTCCCGCCTCTTGTCTTTTCCCATGATTCCTTCCGCACTAGTTTCCCGTGAACCCGAGGACTTCAAACAGCCATACTACAGGCC

-100 CAGGGAAGGCAACCCGCCCCATTAGTTATTATTGGCCAGTCATGGTTAGTCCCGTCCTACATGCTCAGGAAGAGGCGTTTGATTGGTTACGAGCGTGGCC

Gabpa

1 GGAATCTGACGTATTAGGGGGCGTGGCTCAGGCTCAGCTTTCCACCCCCGTACAGCAGCACCATGTACCGCGCACTCCGTCTCCTCGCGCGCTCGCGTCG

YY1

101 CCTCCTGCGGGTTCCGTCCGCCGGTGCTGCAGTGTCTGGGGAAGCGACCACCCTCCCGCGGTGTGCTCCGAACGTCGCGCGAATGGTGAGCGGGCATCTC

201 GGGACCCGGCCTCCCCGGGGTGACCTTCACGTCAGAGTGTCCCGGGTGTCCCCGCGGGCCGCGCCTGGGCTCGGCGCCGGCCAGCACGCTCACCATCCGC

ERR

301 CCCGGACGCGGCCTCCAGCCGAGTGTGTTGGGGCTGCGGGCAGCGCTGAGGGACGCGCTC

Gabpa

# Mdh2

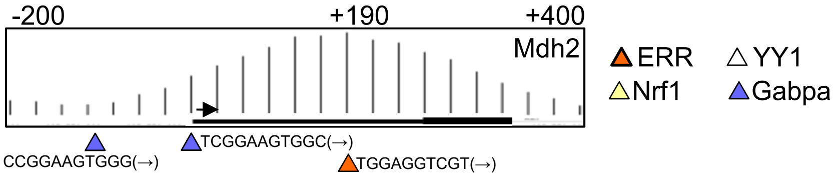


-300 CGCGACTTCTGTCAGGCTCCCCGGCAGGACGTCCGACCGGAGCCCCACCTGGGATAACGGGTACTTCTAACCTAGAGCCTGTCCTCCGGGCGAGCGCTCC

-200 AAGAGTCCTTCTAGTACCGGGTTGCTAGGGGCGGTCCCAAGATTCCGCCTAATCCTGGTGACCGTCTGTCCTAGCGCTAGGTCCCGGAAGTGGGCAAAGC

Gabpa

-100 TAACGGTGGGGAGGGGCGGGGCAAGGGGCGGGGTAGGAGGCGGGGGAAGGAACGGCCTGATAAAGTGAAGCCGGGCCTCTCTCGCGAGATCTCGGAAGTG

Gabpa

1 GCCGGAGCCCAGAGATGACTTCTTTGGAGCCGGAGAGAATCGTGACGTCAGGCCTCGCTCGCCGGGTCTAGGGCGTGCTAGACAAGGAACCACACGGCCG

101 AGCCTGTCTCGCGAGATTTGGCTCAAGCTTCTTGCGCTTCTCTCGCGAGATCAGGTGGCTCTGGACCGGGCGGCCACTGCGCAGAGTGGAGGTCGTTGGA

ERR

201 GTCACTCGTCTTCTTGTAGCTCCTGCCAGTAGCTCCGTGTCCCGCCCGCCCTAGCCATGCTGTCCGCTCTCGCCCGTCCTGCCGGCGCCGCTCTCCGCCG

301 CAGCTTCAGCACTTCGGCCCAGGTACAAGGGGCTGCCTGCCTAGGAAGGAACTCCGGGGGCGGCACACGCGTCCCCGGAGCTCGGCGTTTCTTAACCATC

**Figure S2: Possible binding elements of ERR, YY1, Nrf1, and Gabpa in the genomic sequence of TCA cycle gene promoters.** Genomic localization of PGC-1α (Upper panel) and genomic sequence in TCA cycle gene promoters are shown. The possible transcription factor binding elements are shown as solid arrows with the promoter sequences. TSS is indicated as 1. Dot line indicates degenerative binding elements which was not discovered with an accuracy of 0.85 in JASPAR.

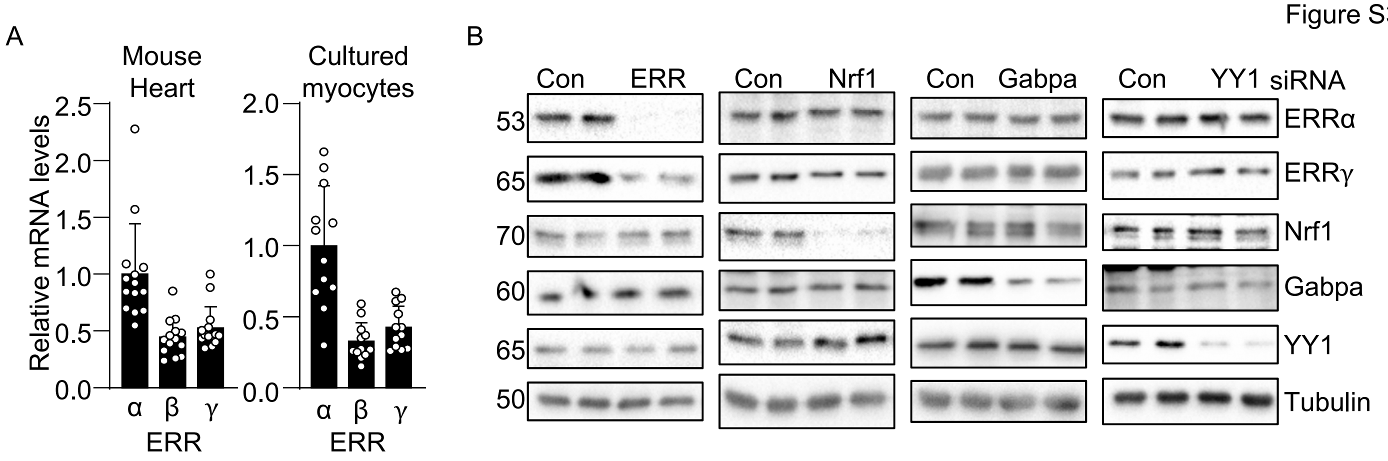


**Figure S3: Knockdown of ERR, Nrf1, Gabpa, and YY1 in cultured cardiomyocytes.** A, The mRNA levels of ERR isoforms in mouse left ventricular (LV) and cultured rat cardiomyocytes. n=12 (Mouse hearts) and 14 (cultured myocytes). B, Knockdown of ERR, Nrf1, Gabpa, and YY1 with short interference RNA. The results were verified with at least 4 biological and 2 technical replicates.

# Cs

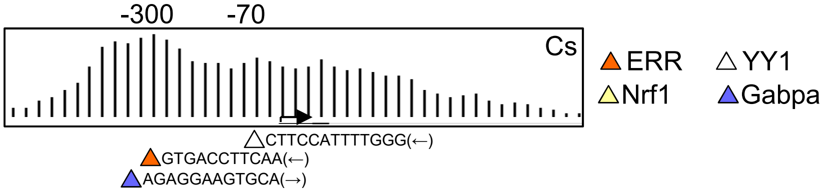


**Mouse**

-400 CGTGATGTCACCTCCACGGCCATGAAGTCATTAACCAAAGACATTCTTTAAGAAAGCATGCCAAGCAGGTAGAGGAAGTGCAATGGTCAGTTGCAGTGTT
Gabpa

-300 CAGTGACCTTCAAACGTCGCGTGCTGGAGCTTTATCTTCAGCATTCTCCGGAGAACCCGAAATGTGTTAAGGGCAAAGGGAAAAGCCAAAGAAGGAAGTT
ERR

-200 AACTGAGTTCTCAGGCACATCCTTAGACACCTACAATCCCTTTCCCACCCACTCCTAATTAGCCTAATCCGTACGGTCCAATCCTCCCCGCGATAACATC

-100 TGTCAAAGGCTCAGATCCCGCCCCTCTTCCATTTTGGGCCAATGGTCGAGGCCGGGGTGGGTCGCCGGAGGGGGGCGGGCTCAAGTTTCGGGCAGAGGGC
YY1

1 GGGGCCTCGCCGAGGACCCTGGGCTGGGCGCCTGTGCCGGTTTGTCTACCCTTCCCCTCAGCCGCCTCCTTTCAACCTTGTCCGCCGGTTGGCGCGGTCT

**Chicken**

-400 GATGTCACCGCAGTGCAGGGCAGTGTCATGGGGTGAGCGCCGCGTGGTGACAACACAGCAGCCCTGAGCCACAGCCGAGCAATAAGGAGGGCACGCGTTG
ERR

-300 GGAGAGAGGGGGGGGGGCCCCCGATGTGGAGGGATGACGTCACGCGGCGATCTGGTGACGTCACGGCGAGTCGGTGACGTCACGGGGCGGGGCTCGCAT

-200 CACCGCACCCCACGCCCAGATCAGGCGAACGCGGAGGCCACCTATAGCCCGGCCCCCCCCGTAGCCTCATTTGCGTAGCCCCGCCCCCATCGGGACAGGG

-100 CTCGGCCAATAGCGAACGGCTCAACCCCGAGGGGGGCGGGCGGGGGCGGGGCTATGCTAATAACGTACGCGGCGCCGCGCCTCGCCCCGCCCGCCGCGCC

1 GAGGACCCCCGGGCGCCGCCGCCGCCGCCGTTCGCTCCGGTCCCGGTCCCGGTCCCGGTCCCGCGCCCGCTCCGTCCGCTCCGCCCCGCGCCGCCATGAC
Nrf1

**Zebrafish**

YY1
-400 AGCACAAATCTGATATTGAGGGGAATTCAGTAATATATTGAAGGTTGATCATTTTAAAGTCTCACATTTATGATGTGTCGACATATTCGGTATTTTTAAA
ERR

-300 ACGATTTAACGTGATCTTACACTTACAAAGTTAACCAAAATACTTAATAAAAAAAACACGTGACTGCATGAGCATATCTGTCATTCTATTTCTAGACAGG

-200 CAGTTAAAAAACCTACAATCCACCATGTACTTATGAATAATTAAGCTACGTTCCATTCGTGAGCCAATCAAATCAAAAAGCGATGAGAGTCTCAACCAAT

-100 CACGTTATTTTTTCTAGTTTTCAACCAATCACATCACGCGATCGCGTTCCATCCTCACCAGGCCCTATCTTACCCAAACCGCGCCTACAACAGGGCCCGA

1 GTCCCTTTTTCAACCTTCACTGCGAGCACGGTAGCGAGAGGGGCACCTTCAAGCAGGGGTCGATAAATAACCACTTGTAAAATTTATACACATATACAAG
YY1 ERR

Figure S4

# Aco2

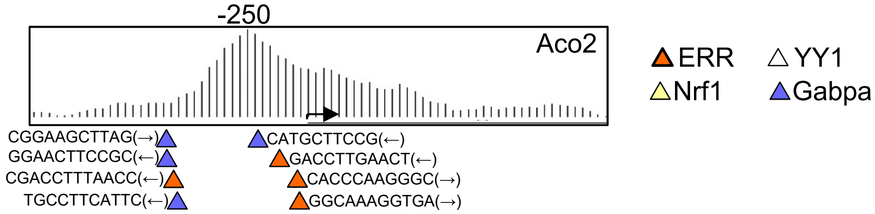


**Mouse**

-500 TCACCCACACCAGCCTGCTTGCGGCAGAAAATCAAATCTGGATGATGTTTAGCCATAACTCTTTTTCCAAGCCGGCGGCCGGAAGCTTAGGGAACTTCCG
Gabpa Gabpa

Gabpa
-400 CCCGACCTTTAACCTACGGCGTGCTTACTTCCGTTCCTCCGTGATTGGCCATTAAGCTGTCCGTCAGCCTTTAGCCTCGGGGGGTGGGGGGGGGCACCAA
ERR

-300 AACCGGCTAGATCTCGCGAGAGTTTCCTCGCTAGGTCCTTGCAGGTGCACCAAAGGTTTTTGAATAAAATTCTCTTTACTTCCAGATTTGAACTCATAAA

-200 CTGAAGTTTGTTCTTGGGTAAAAATTCTACATATGTTGCATTCTGTGTTGGGGATTGACGCCAGGGCTTCTTGCATGCTTCCGCCAAGTATGTTGATTTG
Gabpa

-100 GGGATATTCTCTTTCCTGGATGACCTTGAACTCCTGCCTCCCCTCCAGCTTCCACACCCAAGGGCAAAGGTGACAAGGTTGCGCCGAACGCCGAGTTGGC
ERR ERR ERR

1 AGTTTCCCCGCATCTTTGCTTTCCACGCCGGGTTTCCGTCATGTAAGCCCCGCCCACTGTGCTATCTGCACTCTTGGCTGCGGGAGGAGGCCCCACCCTC

**Chicken**

ERR
-500 CCCGCGCAGCCAAAGCCCCATCCCTGCGCTGGGGAGCGCACGGCGTGCCCTCGGGAAGCGCCACGAACCCCGAGCTCTGAGGGCGGGGAAAGAGCGGGAA
Gabpa Gabpa

-400 GCTACGCGCGCGGGCCCGAACGGCGCCGAACGGGGTCGAACGGCCGCCCGACCCCACCGTCAAGGTGCGTAGGGCTGAGGCCGCGCCGGTACTCACCGAC

-300 GCCCGCCTGCTTGCGGCAGAAGATGAGATCCGGGTGGTGCTTCGCCATGGCGCCGTCGACACTTCTCGGCGCTCTACCGCCGTGCCCCGGTCCTGGCAGG
Gabpa

-200 GGTGTGGAGAGCGTCTGACAAAGCGGCGCTCCGAGAGGGGACACCGACCCGTAACAGAGCTCTGCGTAGTGCTTCAAACTCAGTCTGTCTCGCCGCTCCC

-100 TTCTACCCCGGGAACACCCGTACCCCCACCCGGGGGACGCCGCGTTCTCCGCCCGTACTTCCCTGCCAGGACCCGCTCGGCCCCGCCCCTCGGCGCCCCC

1 ACTCACGCGCCGGAGGTATCAAAAGGACCGACTGACGCCATCGAGGCGCGACTTCATCTTTGTCAGTGCACAAAATGGCGCCGTACTGCGTCCTGGCGGC
Nrf1 YY1

**Zebrafish**

-500 AATACGTCTCCACTAAATTGTGTAAACAGTAAAGAGCTAACATAATATAACAATGTAAAATACTTGTACAACATTTATTCAATTTATTTTAGTTTAAAAC

ERR
-400 ATATATAGACTGTGTGTATTAAATATTGACTTACCAAAGGTTGAGTTCGTTAACATTCATTCATTTATTAATGCATTTGAATAAAGCAAGCAAGTCGGCA
ERR

-300 CATAAATAAAACAGTTATAGGTTATAGACGATAAAAATAGATGTCAGTTTTTGGAAATATCGTTGTCATTTTAGAAAAGGTCCTAAAAGCAAGAGATTAA
YY1

-200 CTCCTGGACAATACTCTACTGGAATCAATTATTAATTTATTCTCCATACCTAAGAGCATACTTTTTGTGTTAGTAATCTATAAGGAAATATCAAAAAAAT

-100 TTAAATGGTCGAATTTTTAAACTTAGGCACGATTAGATTTACCCTCTTCAACAACCTTCCAAGACAACAAGTTCTTATACCCCACACGTCATCTTTTAGC

1 GTCACATCTTTGTCAGTGAACAAAATGGCAACCTACTGCTTAACTGTCGCCCGGCTTCAGGTACATGAAAGTCTGAATTTTATACTGTCGACACGCATGG
YY1 Gabpa

Figure S4

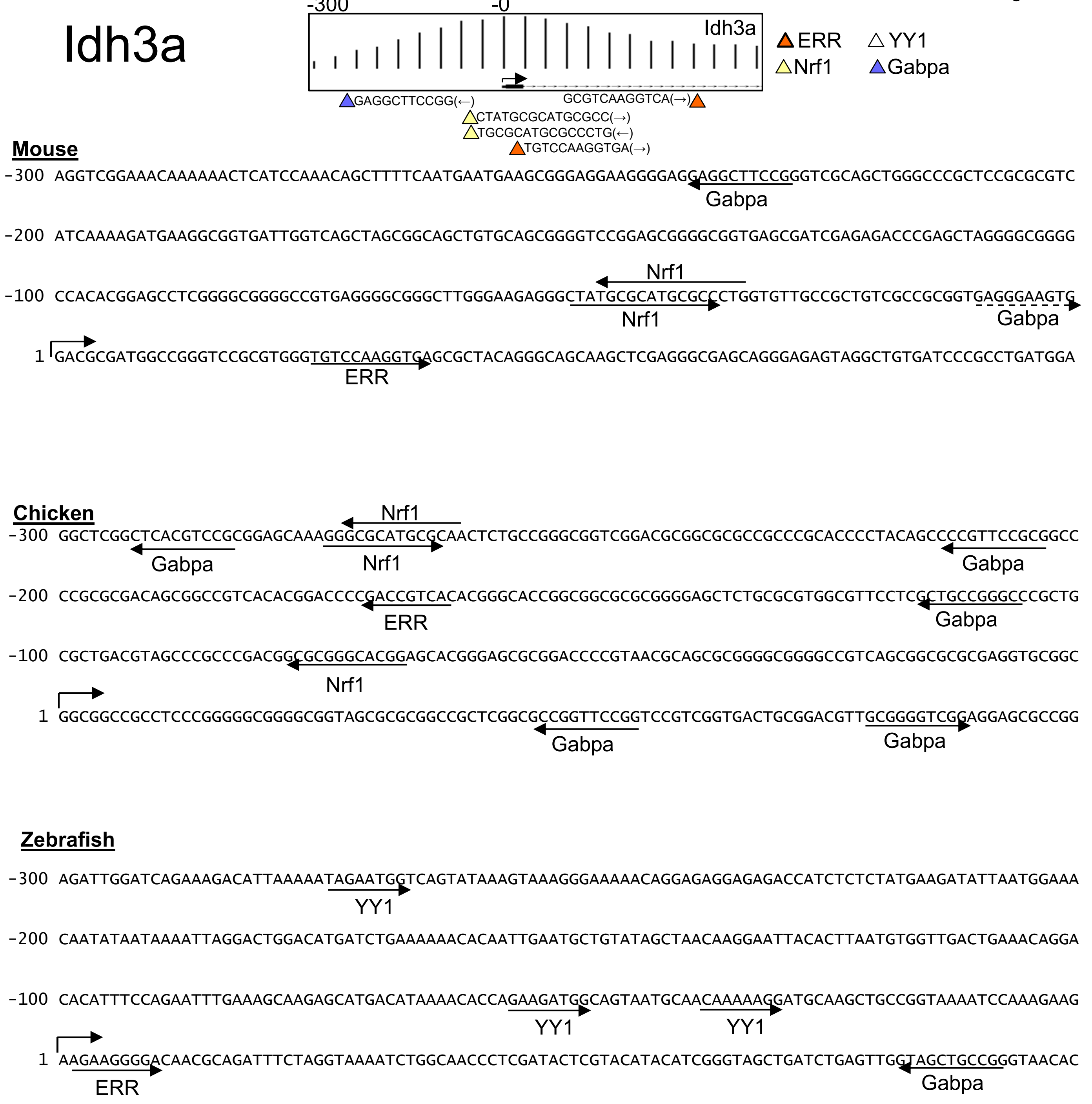


**Figure S4: The TCA cycle gene promoters derived from mouse, chicken, and zebrafish show different sequences.** The transcription factors binding profiling shows different patterns and sequences in Cs, Aco2, and Idh3a promoters.

Figure S5

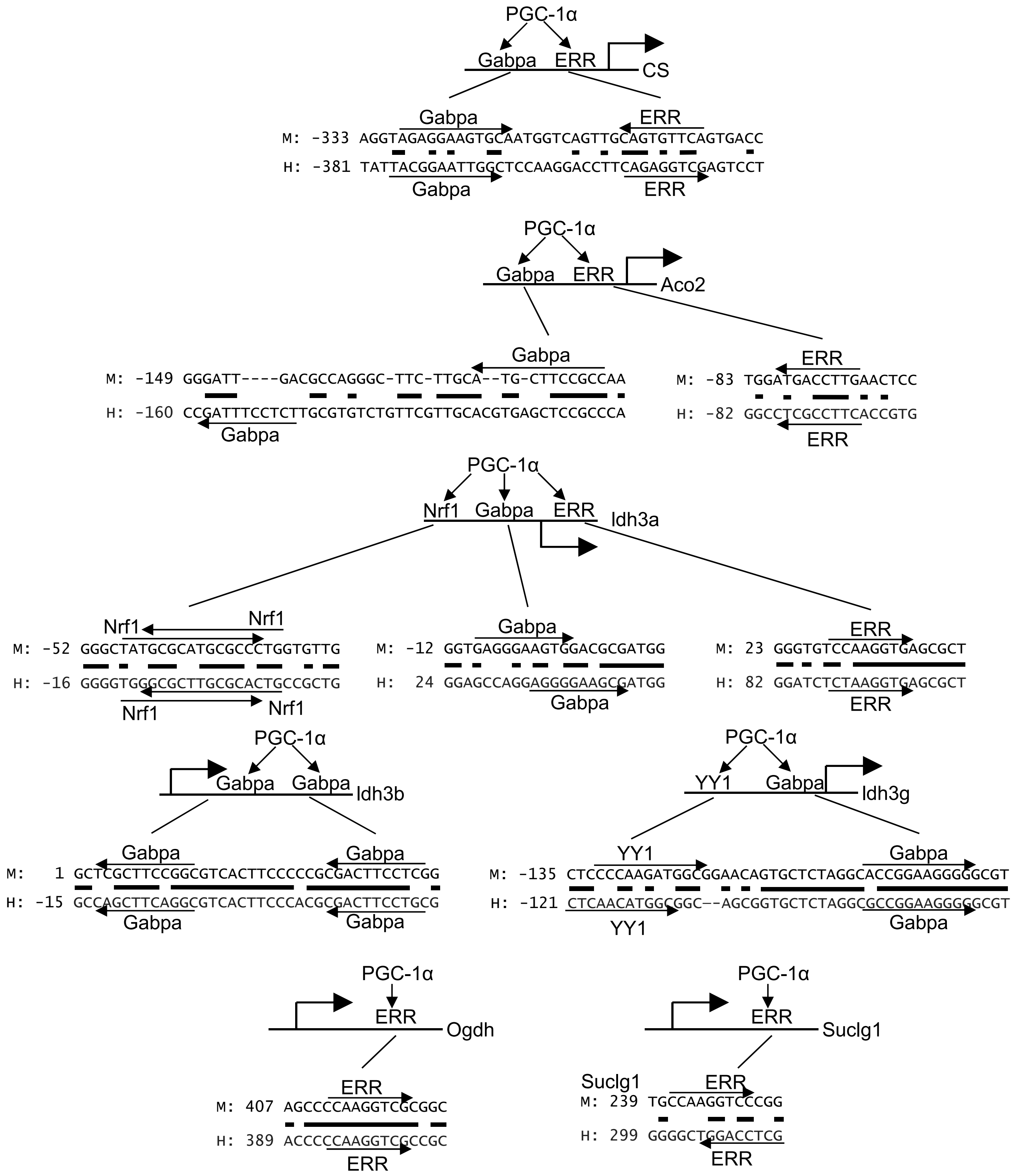

PGC-1α
Gabpa
ERR
CS
M: -333 AGGTAGAGGAAGTGCAATGGTCAGTTGCAGTGTTCAGTGACC
H: -381 TATTACGGAATTGGCTCCAAGGACCTTCAGAGGTCGAGTCCT
Aco2
M: -149 GGGATT----GACGCCAGGGC-TTC-TTGCA--TG-CTTCCGCCAA
H: -160 CCGATTTCCTCTTGCGTGTCTGTTCGTTGCACGTGAGCTCCGCCCA
M: -83 TGGATGACCTTGAACTCC
H: -82 GGCCTCGCCTTCACCGTG
Nrf1
Idh3a
M: -52 GGGCTATGCGCATGCGCCCTGGTGTTG
H: -16 GGGGTGGGCGCTTGCGCACTGCCGCTG
M: -12 GGTGAGGGAAGTGGACGCGATGG
H: 24 GGAGCCAGGAGGGGAAGCGATGG
M: 23 GGGTGTCCAAGGTGAGCGCT
H: 82 GGATCTCTAAGGTGAGCGCT
Idh3b
M: 1 GCTCGCTTCCGGCGTCACTTCCCCCGCGACTTCCTCGG
H: -15 GCCAGCTTCAGGCGTCACTTCCCACGCGACTTCCTGCG
YY1
Idh3g
M: -135 CTCCCCAAGATGGCGGAACAGTGCTCTAGGCACCGGAAGGGGGCGT
H: -121 CTCAACATGGCGGC--AGCGGTGCTCTAGGCGCCGGAAGGGGGCGT
Ogdh
M: 407 AGCCCCAAGGTCGCGGC
H: 389 ACCCCCAAGGTCGCCGC
Suclg1
M: 239 TGCCAAGGTCCCGG
H: 299 GGGGCTGGACCTCG

Figure S5

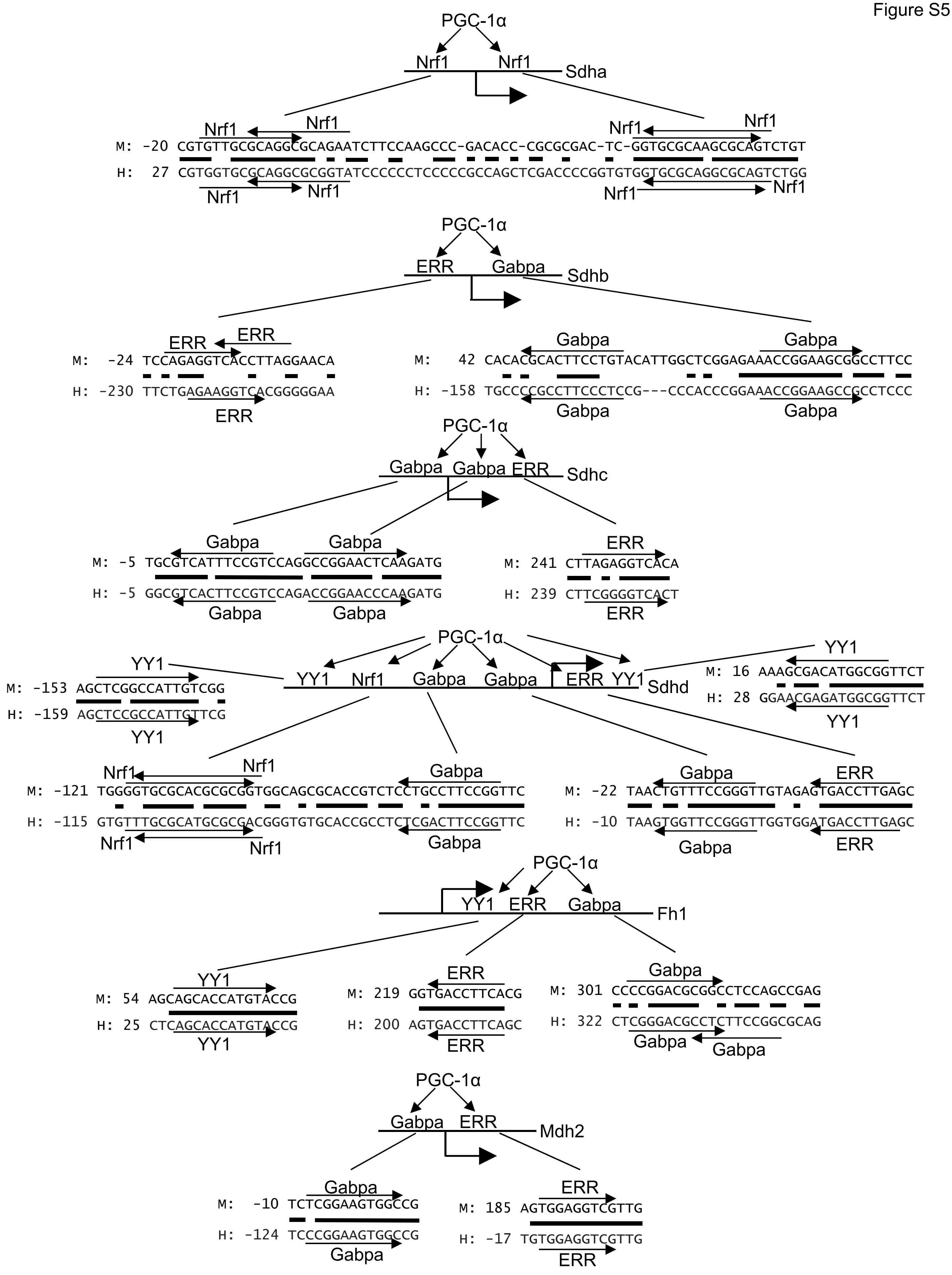

PGC-1α
Nrf1
Nrf1
Sdha
M: -20 CGTGTTGCGCAGGCGCAGAATCTTCCAAGCCC-GACACC-CGCGCGAC-TC-GGTGCGCAAGCGCAGTCTGT
H: 27 CGTGGTGCGCAGGCGCGGTATCCCCCCTCCCCGCCAGCTCGACCCCGGTGTGGTGCGCAGGCGCAGTCTGG
PGC-1α
ERR
Gabpa
Sdhb
M: -24 TCCAGAGGTCACCTTAGGAACA
H: -230 TTCTGAGAAGGTCACGGGGGAA
M: 42 CACACGCACTTCCTGTACATTGGCTCGGAGAAACCGGAAGCGGCCTTCC
H: -158 TGCCCCGCCTTCCCTCCG---CCCACCCGGAAACCGGAAGCCGCCTCCC
PGC-1α
Gabpa
Gabpa
ERR
Sdhc
M: -5 TGCGTCATTTCCGTCCAGGCCGGAACTCAAGATG
H: -5 GGCGTCACTTCCGTCCAGACCGGAACCCAAGATG
M: 241 CTTAGAGGTCACA
H: 239 CTTCGGGGTCACT
PGC-1α
YY1
Nrf1
Gabpa
Gabpa
ERR
YY1
Sdhd
M: -153 AGCTCGGCCATTGTCGG
H: -159 AGCTCCGCCATTGTTCG
M: 16 AAAGCGACATGGCGGTTCT
H: 28 GGAACGAGATGGCGGTTCT
M: -121 TGGGGTGCGCACGCGCGGTGGCAGCGCACCGTCTCCTGCCTTCCGGTTC
H: -115 GTGTTTGCGCATGCGCGACGGGTGTGCACCGCCTCTCGACTTCCGGTTC
M: -22 TAACTGTTTCCGGGTTGTAGAGTGACCTTGAGC
H: -10 TAAGTGGTTCCGGGTTGGTGGATGACCTTGAGC
PGC-1α
YY1
ERR
Gabpa
Fh1
M: 54 AGCAGCACCATGTACCG
H: 25 CTCAGCACCATGTACCG
M: 219 GGTGACCTTCACG
H: 200 AGTGACCTTCAGC
M: 301 CCCCGGACGCGGCCTCCAGCCGAG
H: 322 CTCGGGACGCCTCTTCCGGCGCAG
PGC-1α
Gabpa
ERR
Mdh2
M: -10 TCTCGGAAGTGGCCG
H: -124 TCCCGGAAGTGGCCG
M: 185 AGTGGAGGTCGTTG
H: -17 TGTGGAGGTCGTTG

**Figure S5: Schematic representation of the critical transcription factor binding elements in mice and humans.** The transcription factors 1) whose binding element is found proximal to a PGC-1α localization peak and 2) whose knockdown suppresses the genes are indicated. Number indicates distance from TSS. Corresponding elements in the humans are also indicated.

Figure S6

Figure S3B

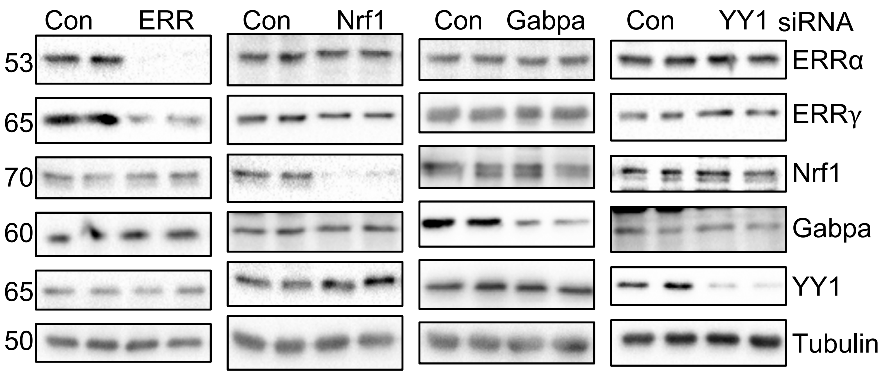


Western blot images with molecular weight markers

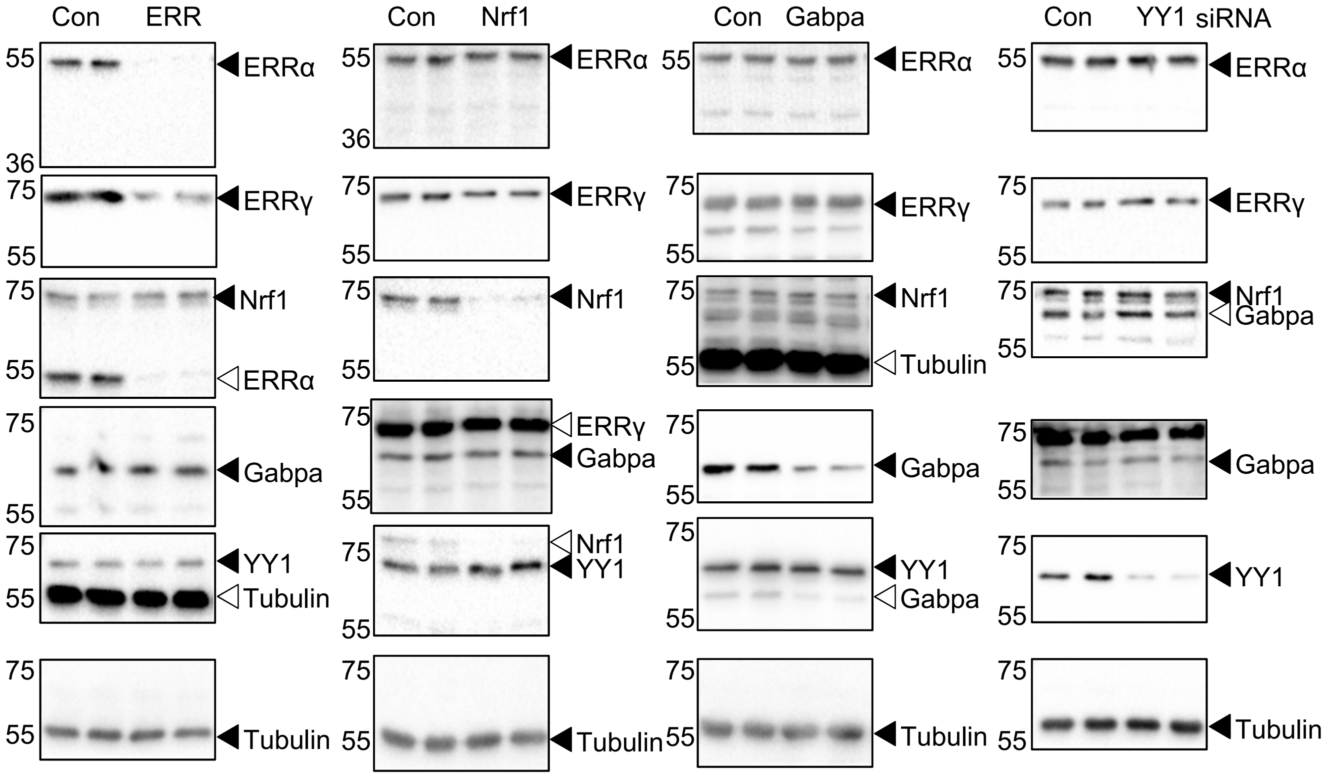

Figure S6

Figure 6B

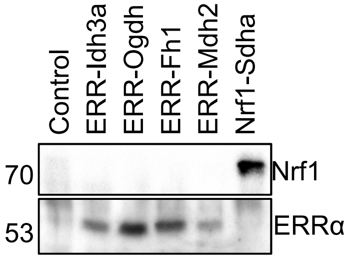


Figure 6C

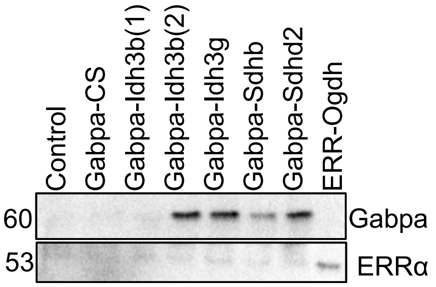


Western blot images with molecular weight markers

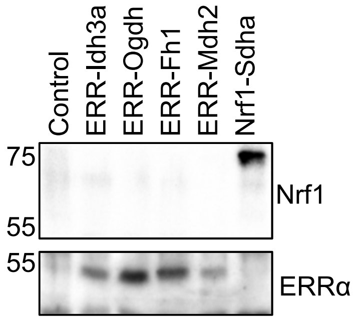


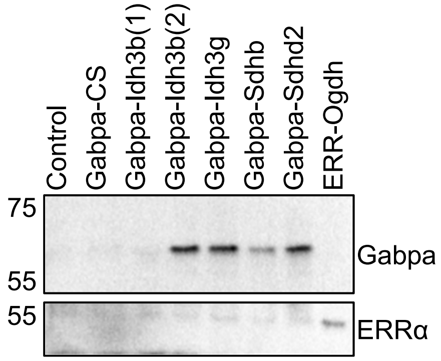

Figure S6

Figure 6E

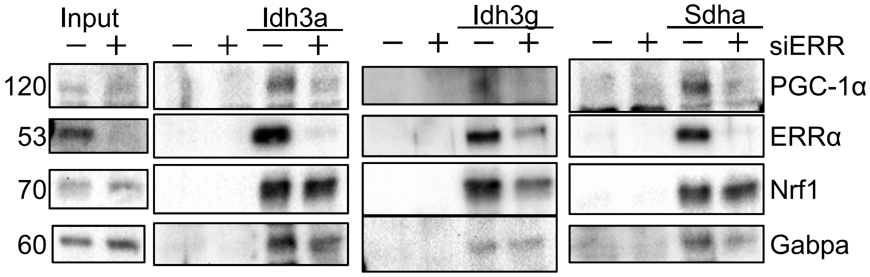


Western blot images with molecular weight markers

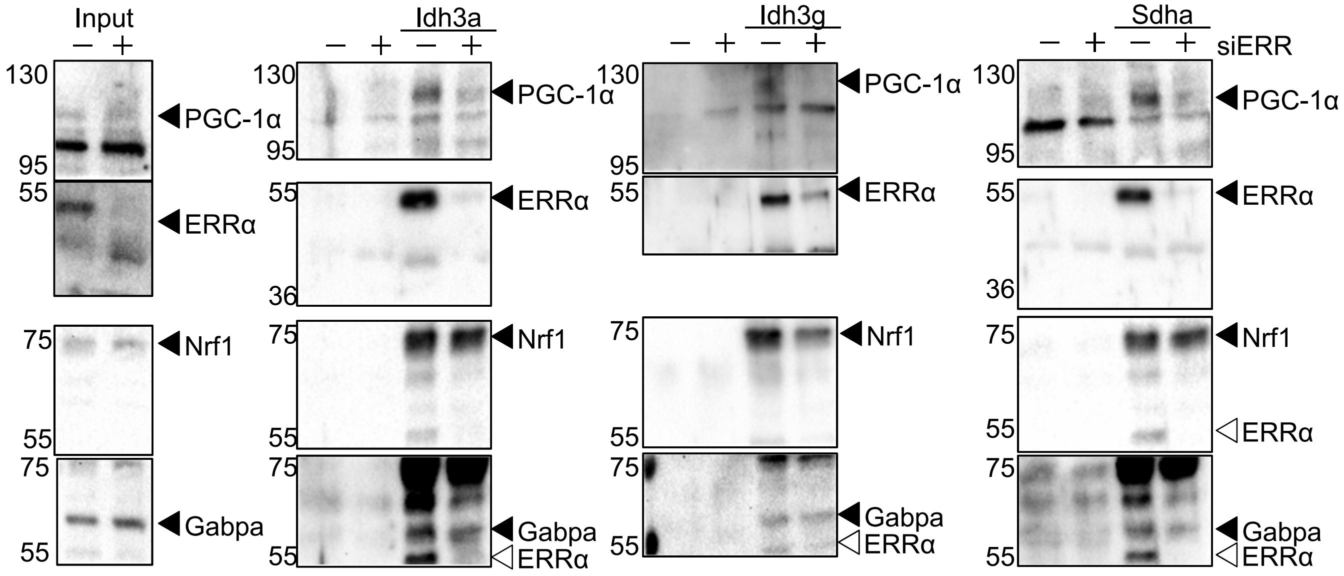

Figure S6

Figure 6E

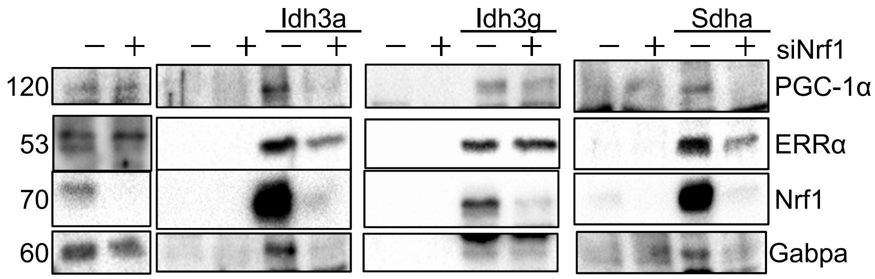


Western blot images with molecular weight markers

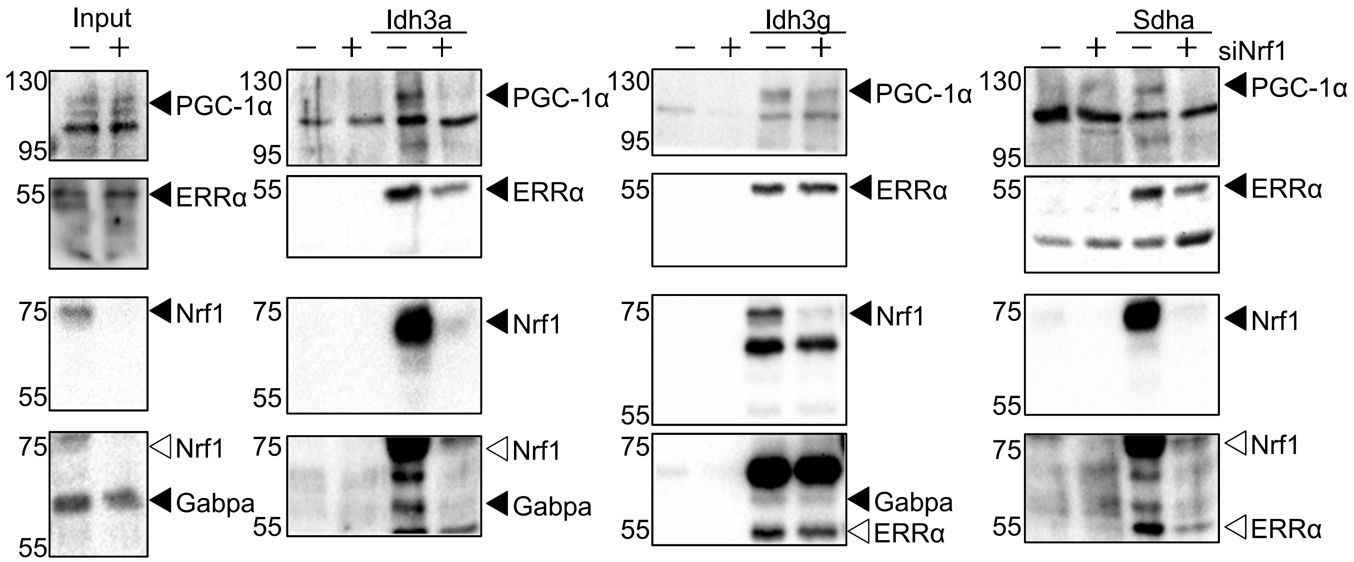

Figure 6E

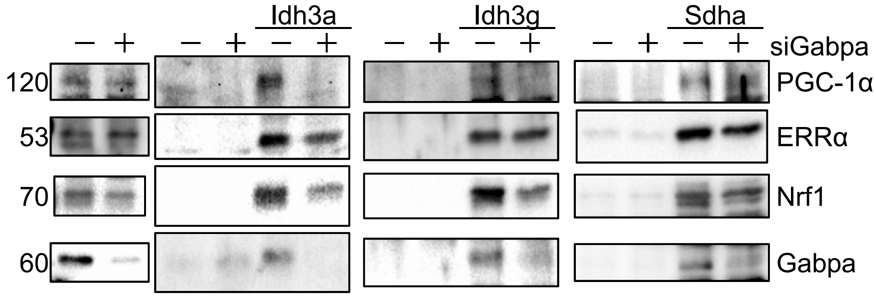


Western blot images with molecular weight markers

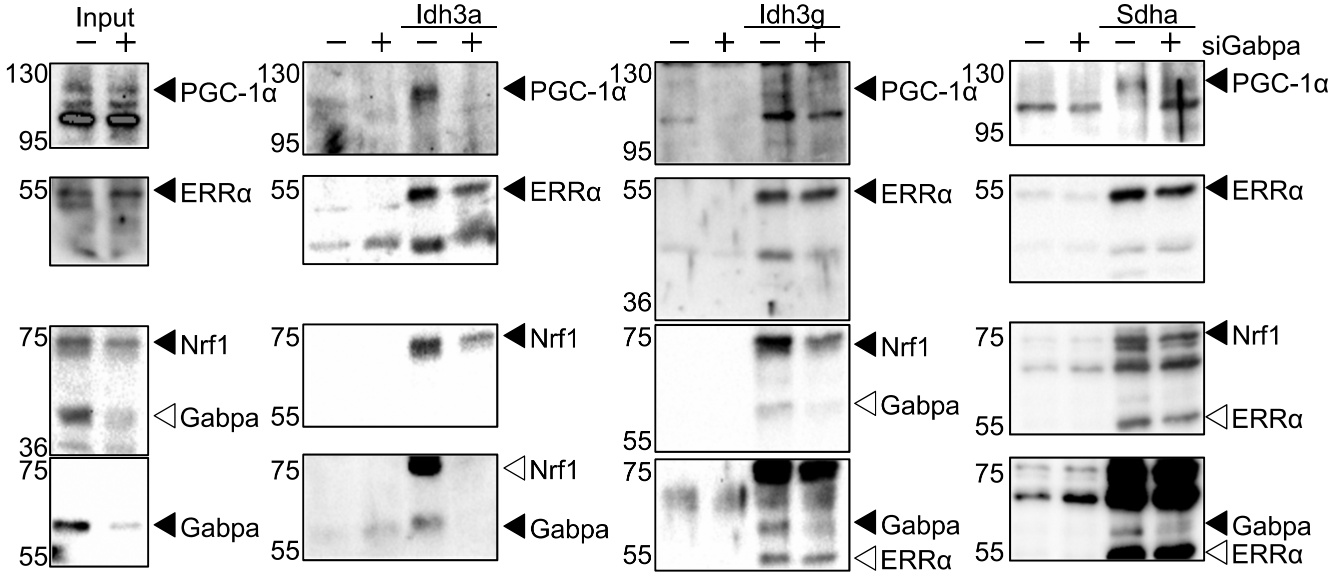

Figure S7B

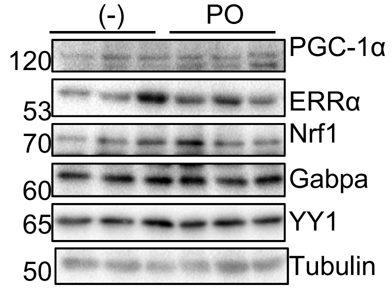


Western blot images with molecular weight markers

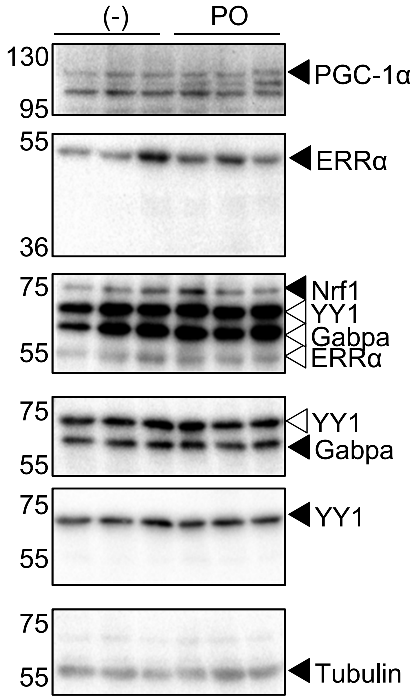

Figure S6

Figure 7C

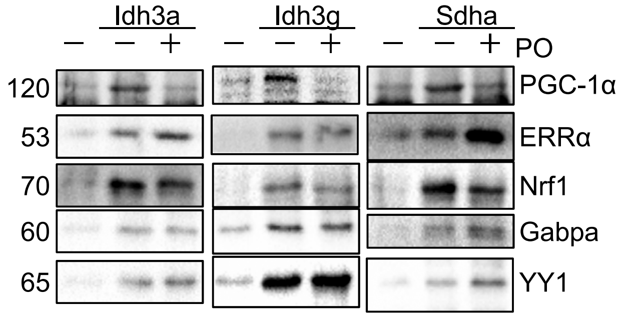


Western blot images with molecular weight markers

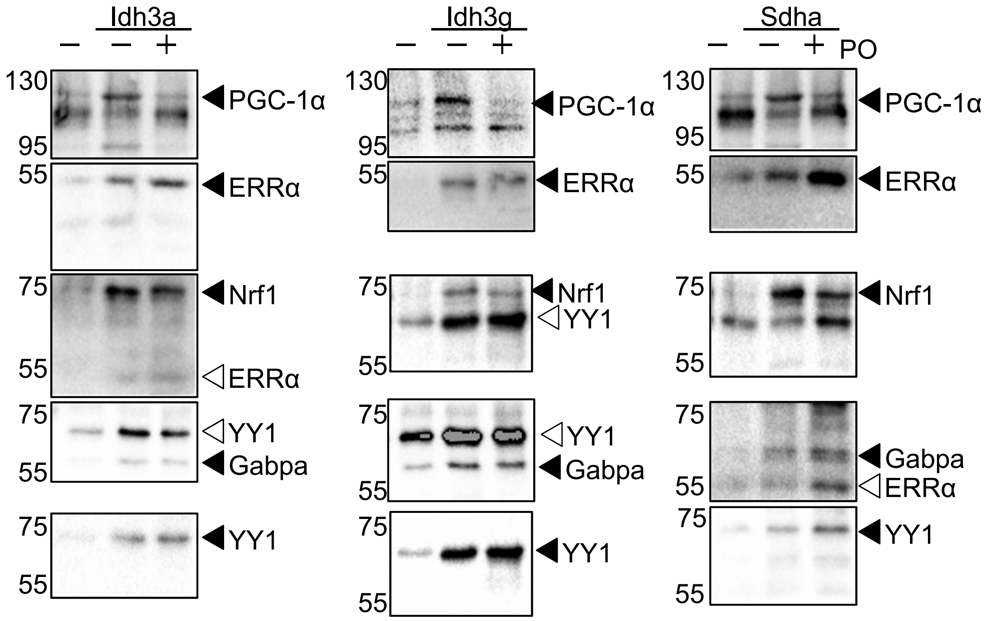


**Figure S6: Immunoblot images with molecular weight markers.** Filled arrow indicates specific signal for the blot, whereas opened arrow indicates left over signal from prior immunoblots.
